# Exploring the role of cyclodextrins as a cholesterol scavenger: a molecular dynamics investigation of conformational changes and thermodynamics

**DOI:** 10.1038/s41598-023-49217-8

**Published:** 2023-12-08

**Authors:** Mokhtar Ganjali Koli, Federico Fogolari

**Affiliations:** 1 Department of Chemistry, University of Kurdistan, Sanandaj, Iran; 2 Computational Chemistry Laboratory, Kask Afrand Exire Ltd., Sanandaj, Iran; 3 Dipartimento di Scienze Matematiche Informatiche e Fisiche (DMIF), University of Udine, Via delle Scienze 206, 33100 Udine, Italy

**Keywords:** Biophysical chemistry, Computational chemistry, Molecular dynamics

## Abstract

This study presents a comprehensive analysis of the cholesterol binding mechanism and conformational changes in cyclodextrin (CD) carriers, namely βCD, 2HPβCD, and MβCD. The results revealed that the binding of cholesterol to CDs was spontaneous and thermodynamically favorable, with van der Waals interactions playing a dominant role, while Coulombic interactions have a negligible contribution. The solubility of cholesterol/βCD and cholesterol/MβCD complexes was lower compared to cholesterol/2HPβCD complex due to stronger vdW and Coulombic repulsion between water and CDs. Hydrogen bonding was found to have a minor role in the binding process. The investigation of mechanisms and kinetics of binding demonstrated that cholesterol permeates into the CD cavities completely. Replicas consideration indicated that while the binding to 2HPβCD occurred perpendicularly and solely through positioning cholesterol's oxygen toward the primary hydroxyl rim (PHR), the mechanism of cholesterol binding to βCD and MβCD could take place with the orientation of oxygen towards both rims. Functionalization resulted in decreased cavity polarity, increased constriction tendency, and altered solubility and configuration of the carrier. Upon cholesterol binding, the CDs expanded, increasing the cavity volume in cholesterol-containing systems. The effects of cholesterol on the relative shape anisotropy (*κ*^2^) and asphericity parameter (*b*) in cyclodextrins were investigated. βCD exhibited a spherical structure regardless of cholesterol presence, while 2HPβCD and MβCD displayed more pronounced non-sphericity in the absence of cholesterol. Loading cholesterol transformed 2HPβCD and MβCD into more spherical shapes, with increased probabilities of higher *κ*^*2*^. MβCD showed a higher maximum peak of *κ*^*2*^ compared to 2HPβCD after cholesterol loading, while 2HPβCD maintained a significant maximum peak at 0.2 for *b*.

## Introduction

Cholesterol is a lipid molecule that plays a crucial role in various biological processes, including membrane structure, cell signaling, and hormone synthesis. However, an excess of cholesterol in the blood can lead to the development of cardiovascular diseases, such as atherosclerosis, which is a leading cause of morbidity and mortality worldwide^1^. Therefore, controlling cholesterol levels in the body is of utmost importance for maintaining overall health. One promising approach for cholesterol control is the use of cyclodextrins (CDs), which are cyclic oligosaccharides composed of glucose units. CDs can form inclusion complexes with cholesterol, in which the cholesterol molecule is trapped inside the CD cavity. This complexation results in enhancing the solubility of cholesterol, which can facilitate its removal from the body^2–4^. The interaction between cholesterol and CDs has been extensively studied in both in vitro and in vivo settings^5–8^. In vivo studies have also shown the potential of CDs as cholesterol-lowering agents. It well proven that β-Cyclodextrin (βCD) effectively prevents cholesterol gallstone formation in LPN hamsters by reducing the reabsorption of chenodeoxycholate, stimulating its biosynthesis and promoting its fecal elimination^4^. It suggests that βCD could be a promising therapeutic option for preventing cholesterol gallstones. The effect of dietary βCD on the cholesterol of blood and tissues of swine was investigated. It was shown that addition of βCD to the basal diets containing 1.5%, 3.0%, or 5.0% βCD significantly reduced total lipid, triglyceride and total cholesterol levels in swine blood, especially in the group receiving 5.0% βCD-fed^9^. Methylated-βCDs (MβCDs) can reduce the cellular cholesterol content of cells involved in atherosclerotic lesions and modulate the expression of ABC transporters involved in reverse cholesterol transport. The use of MβCDs could be a promising therapeutic tool to interfere with atherosclerosis pathogenesis in patients^10^. Studies have demonstrated that HDL and βCDs can extract cholesterol from aggregated LDL^5,11^. HDL could aid in removing cholesterol from lesions and injecting hydroxypropyl-β-cyclodextrin (HPβCD) can promote regression of atherosclerotic lesions in mice^12^. HPβCD has also been found effective in removing cholesterol deposits from atherosclerotic plaques and reduced plasma triglyceride levels and inflammatory cytokine levels in rabbits^13^. In the food industry, the use of βCD is a successful method for eliminating cholesterol from milk, achieving a rate of up to 98%. This approach does not significantly impact the nutritional, textural, or flavor qualities of the final product, and it is also easy to apply, effective, low in cost, and feasible on current production lines^14^. The mechanisms underlying these interactions are still being investigated, but the potential applications of CDs as cholesterol-lowering agents have been demonstrated in preclinical and clinical studies. The use of CDs as cholesterol-lowering agents has the potential to provide a safe and effective alternative to traditional cholesterol-lowering drugs^15,16^. The interaction between cholesterol and CDs is primarily driven by the hydrophobic effect, which arises from the tendency of nonpolar molecules to aggregate in aqueous solutions. CDs have a hydrophobic cavity that can encapsulate cholesterol molecules, shielding them from the aqueous environment^17^. The formation of cholesterol-CDs complex could be confirmed by phase solubility studies, Fourier transform infrared spectroscopy (FTIR), X-ray diffractometry, and differential scanning calorimetry (DSC)^18^. Meanwhile, computational methods such as molecular dynamics simulations is among the most powerful tools for studying biological systems^19–25^. In this investigation, molecular dynamics (MD) simulations were employed to offer an elucidation of the thermodynamic and dynamic aspects concerning the complexes formed between cholesterol and cyclodextrins (CDs). By delving into the underlying mechanisms governing their interactions, we sought to pave the way for the development of more efficacious cholesterol-CD systems. Our comprehensive exploration encompassed a diverse array of properties, including the binding affinity, orientation, dynamics of cholesterol encapsulated within the CD cavity, and structural modifications of the CD molecule. The primary objective of this study was to scrutinize the mutual influences between cholesterol-CD interactions, aiming to attain an in-depth comprehension of the dynamic and thermodynamic processes underlying the creation of host–guest systems involving a Cholesterol molecule and various CD derivatives, such as β-Cyclodextrin (βCD), 2-Hydroxypropyl-βCD (2HPβCD), and Heptakis(2,6-di-O-methyl)-βCD (MβCD).

## Computational details

### Molecular dynamics simulation

**Figure 1:**
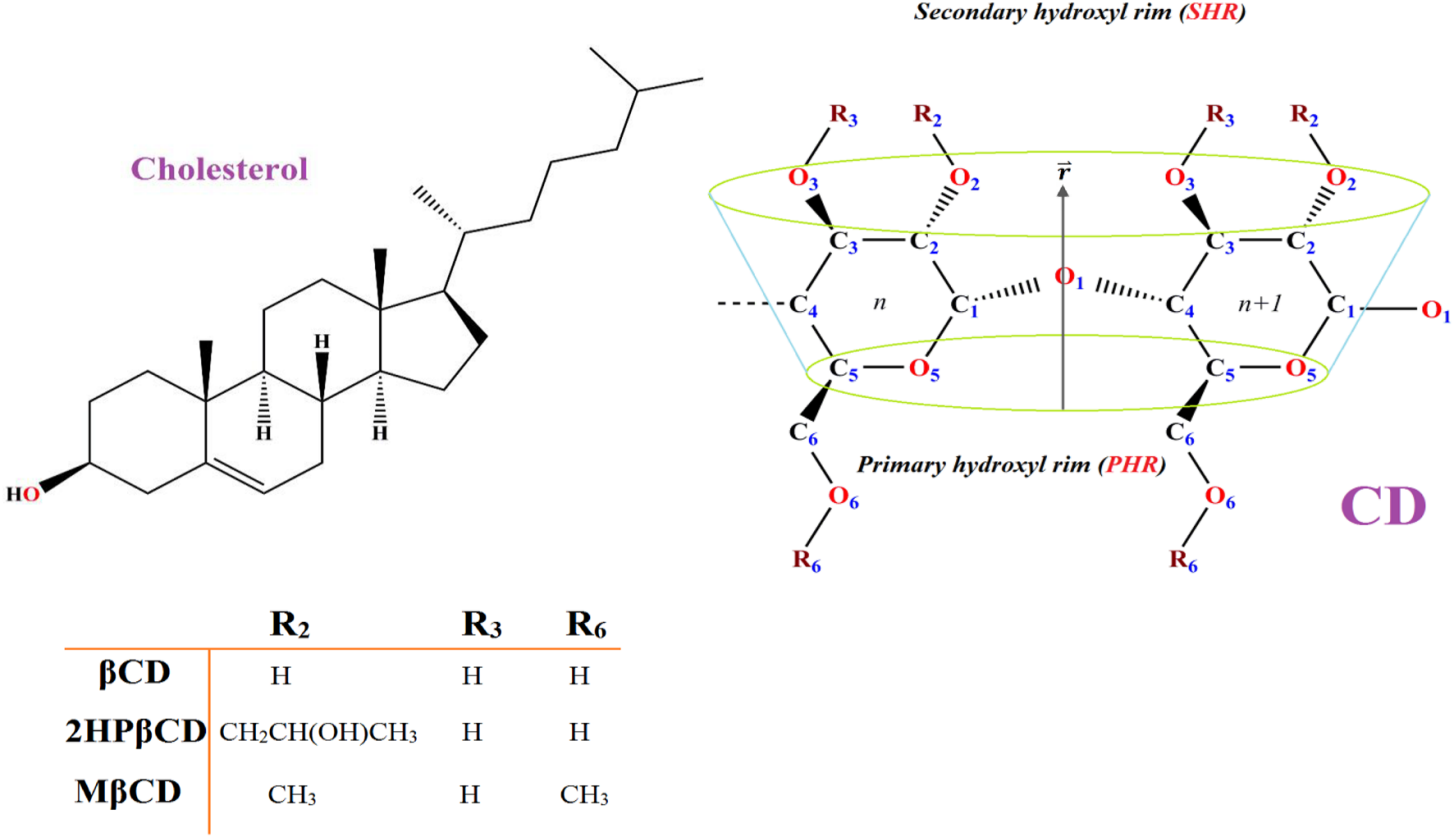
Molecular structure of βCD derivatives and cholesterol.

Figure 1 and Fig. S1 illustrate the molecular structures of cholesterol and CDs. In total, seven distinct systems were created and exposed to simulation. The first three systems, considered as references, each consisted of individual CDs and water molecules. The next three systems were composed of a cholesterol molecule, water, and one of the CDs. The final system consisted solely of a cholesterol molecule and water. All simulations were conducted utilizing the GROMACS 2020 software package^26,27^ in the NPT ensemble. The GROMOS 54a7 force field was employed for the representation of the uncharged state of CD molecules and cholesterol^28,29^, a force field whose parameters for CD molecule have been validated in previous studies^21^.

**Figure 2:**
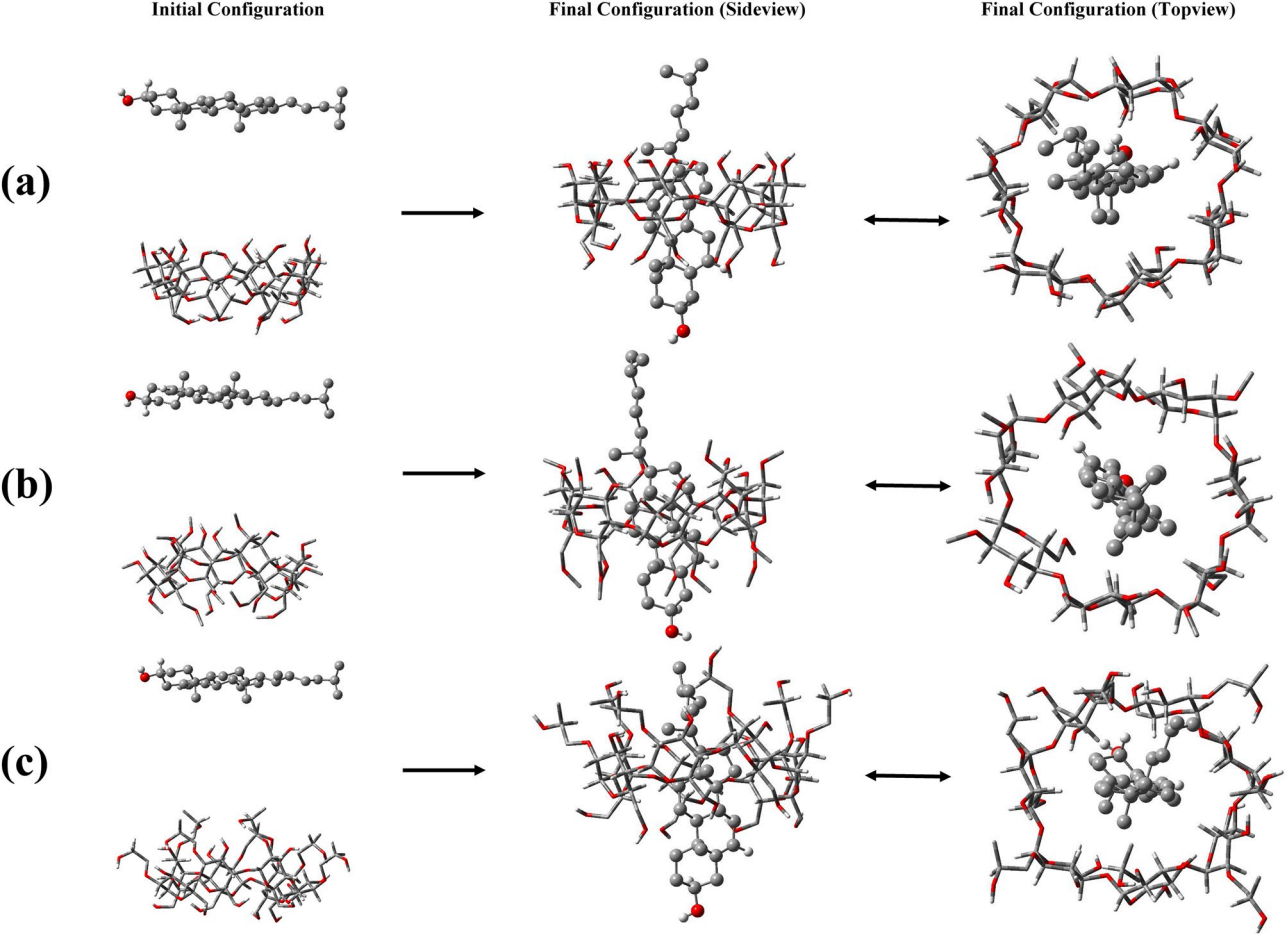
Snapshots of the initial and final configurations of cholesterol toward βCD (**a**), MβCD (**b**), and 2HPβCD (**c**).

To avoid any imposing interactions between the cholesterol and CDs molecules, the initial configurations were set up such that the Cholesterol was approximately 1.5 nm away from the secondary hydroxyl rim (SHR), as depicted in Fig. 2. For each system involving cholesterol and CD, four additional replicas were considered by altering the position of cholesterol. This alteration placed cholesterol at a 180° angle from the SHR to the primary hydroxyl rim (PHR), encompassing all possible configurations, as shown in Fig. S2. Each system was provided with 5000 water molecules for hydration, and periodic boundary conditions were enforced. The water molecules were represented using the extended simple point charge model (SPC/E)^30^. To eliminate unfavorable atomic interactions, a steepest descent energy minimization was employed^31^, and the systems underwent equilibration processes within the NVT and NPT ensembles for 1 ns and 9 ns, respectively. After achieving equilibration, the simulations were conducted over 200 ns, with the maintenance of bond length constraints facilitated by the LINCS algorithm^32^. The only exception was related to Replica-3 of 2HPβCD, in which the simulation time extended to 370 ns. This longer time was due to the occurrence of complete loading just after 318 ns, unlike the other three replicas. This suggests that the accurate sampling of the phase space (the space of all possible configurations) in this configuration is longer than the others.

The temperature was held at 310 K through the application of the V-rescale coupling technique^33^, employing a time constant of 0.1 ps, while the pressure was kept at 1 bar via the utilization of the Parrinello-Rahman barostat^34^ with a coupling time constant of 2 ps. The integration of Newton’s motion equations was accomplished utilizing the leap-frog algorithm with a time step of 2 fs^35^. The simulation employed the particle mesh Ewald (PME) method^36^ to address long-range Coulombic interactions, and a cut-off radius of 1.2 nm was implemented for both Coulombic and van der Waals (vdW) interactions. In this research, GaussView 5.0 software was employed to visualize the geometries^37^, while two-dimensional (2D) chemical structures were created using ChemDraw Professional 16.

### Free energy computations

The solvation free energy of a solvated system was determined by evaluating the free energy difference in a transforming system as it moves between solvated and unsolvated states. This computational analysis involved the utilization of a coupling parameter (λ) in conjunction with the thermodynamic integration (TI) formula^38,39^.

1 $${\Delta G}_{AB}={\int }_{{\uplambda }_{A}}^{{\uplambda }_{B}}{\langle \frac{\partial H (\uplambda )}{\partial\uplambda }\rangle }_{{\uplambda}^{\prime}}d\uplambda $$

The Hamiltonian (H) was quantified as a function of the coupling parameter λ. This parameter serves as an indicator of the extent of transformation occurring between specific states, such as the transition from solvated to unsolvated states or between states A and B. The TI method relies on the λ-dependence of the Hamiltonian for solute–solvent interactions, which gradually varies between full interactions (corresponding to λ = 0) and no interaction (corresponding to λ = 1). In other words, the solute molecule is gradually made to disappear from the solution using the coupling parameter λ. The Bennett acceptance ratio method (BAR)^40^ was utilized to determine the differences in free energy between Hamiltonians at various λ values. A thermodynamic cycle that relied on alchemical free energy calculations^41^ was employed to evaluate the binding free energy (see more details on page 5 in the Supplementary Information). The computation of the free energy associated with the interaction between the cholesterol and its surroundings involved gradually reducing the potential energy of interaction between the cholesterol molecule and their environment using a λ parameter, which ranged from 0 to 1^42^. To calculate ΔG_solv_, a total of 25 λ points were used. Initially, Coulombic solute–solvent interactions were turned off at a larger λ value compared to vdW terms^43^ in order to avoid unstable Coulombic interactions that could lead to unreliable energies and unstable configurations. Soft-core potentials with specific parameters (α = 0.5, σ = 0.3, and p = 1)^42,44^ were implemented to prevent the overlap of atoms between the solute and solvent at low λ values. The starting point for the free energy calculations was obtained from the final configuration of each simulated system, and each simulation had a duration time of 5 ns.

## Results and discussion

### Binding mechanism and orientation of cholesterol

**Figure 3:**
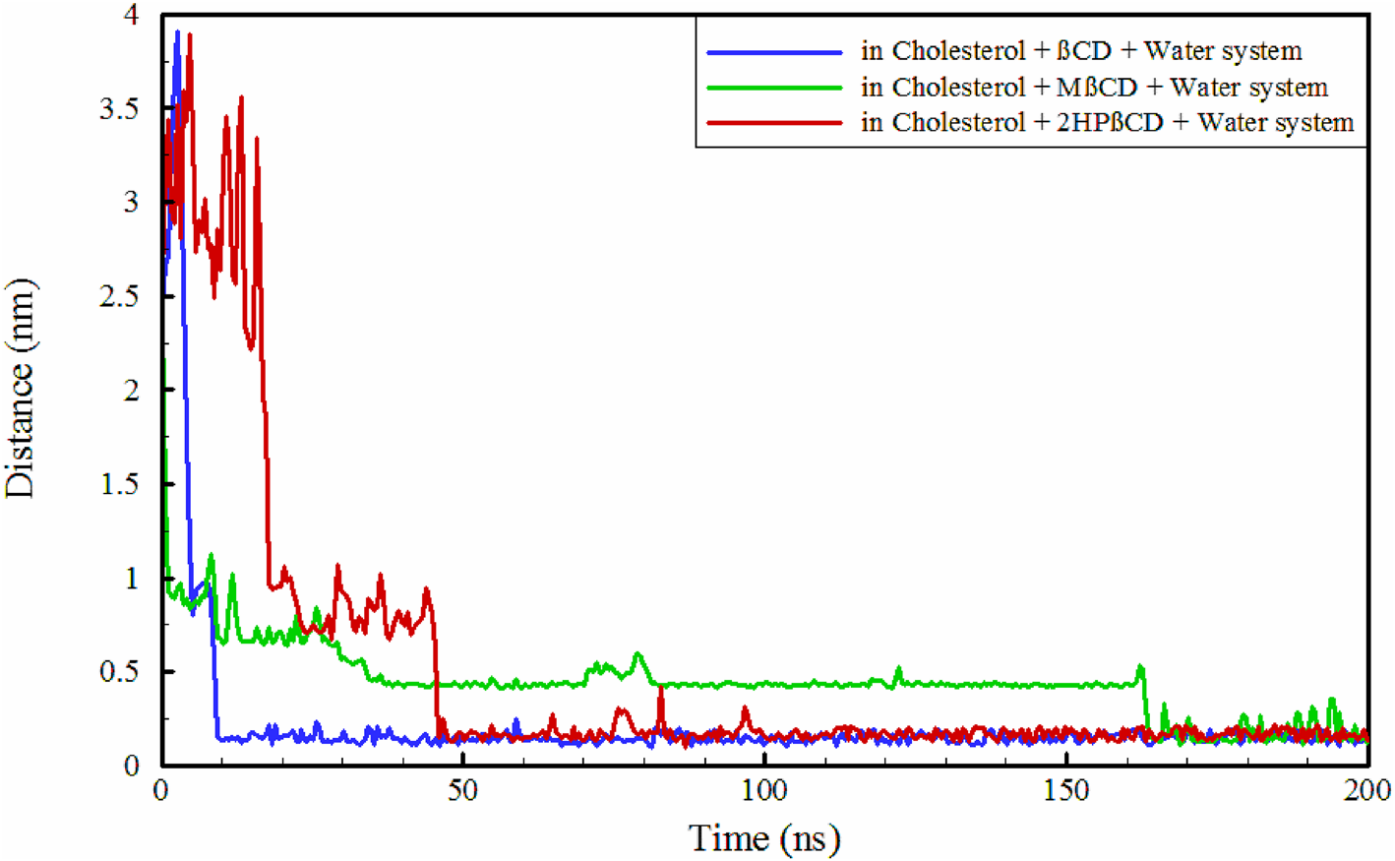
Time evaluation the distance between cholesterol and the center of CD molecules.

An essential component of understanding the dynamics of guest molecules within CDs is the extent to which they can permeate the central region of the CD molecules. Loading a guest molecule into CDs offers numerous benefits such as enhanced solubility, protection, controlled release, modified properties, and increased bioavailability^45,46^. The initial and final configurations of all cholesterol-containing systems are depicted in Fig. 2. Furthermore, the final configuration of the replicas was also extracted at the last 10% of the simulation time, as illustrated in Figure S3, S4, S5. As can be seen, cholesterol has fully and vertically penetrated into the cavity of βCD and MβCD with two possible mechanisms and binding could occur in such a way that the oxygen atom of cholesterol is positioned towards both the SHR and PHR. Figure 3 and Fig. S6 illustrate the distances between the center of geometry (COG) of cholesterol and the CD molecules. As observed, cholesterol rapidly binds to MβCD, whereas its binding to βCD takes slightly longer and only occurs after approximately 5 ns from the beginning of the simulation. Through trajectory analysis, it was determined that the binding of cholesterol to MβCD did not occur in a vertical manner; instead, it happened horizontally, positioned on the surface of the SHR. This configuration remained stable and unchanged for approximately 160 ns. Only after this period, the position of the cholesterol relative to MβCD changed, as it was placed vertically within MβCD's cavity, as seen in Fig. 3. Finally, cholesterol binds to 2HPβCD with a significantly longer time interval, around 20 ns. In comparison to βCD and 2HPβCD, despite the faster binding kinetics of cholesterol to MβCD, it should be noted that due to the fact that cholesterol does not fully penetrate into the cavity, it is more prone to exposure to environmental factors. Moreover, it appears that the oxygen atom of cholesterol is located outside the cavity of CDs. This observation is due to the hydrophilic nature of oxygen, which consequently tends to interact with water.

### Penetration of water molecules

In the absence of a guest molecule, it is common for water molecules to typically fill the CD cavity within an aqueous solution. Extensive analysis using X-ray and neutron diffraction techniques has provided evidence that the cavities of α-CD, β-CD, and γ-CD contain an average of 2.6, 6.5, and 8.8 water molecules, respectively. These water molecules are distributed among several locations within the cavity^47–52^. These confined waters fail to form a complete hydrogen bond network as they would in a bulk of water, resulting in a high-energy state. Upon the entrance of a guest into the cavity space, these high-energy water molecules are released, facilitating the formation of the cavity-guest complex from a thermodynamic perspective within the cavity^53–56^. Therefore, by considering spheres with various radii within the central region of CD cavities, the number of water molecules in different layers was specified, as illustrated in Table 1 and Table S1-S3. The first tangible finding in this regard is that as a result of the functionalization of βCD and the formation of 2HβCD and MβCD, the number of water molecules inside the cavity decreases significantly. Specifically, within the innermost layer (0–0.5 nm), the number of water molecules decreases dramatically from 9.56 in βCD to 0.9 and 1.56 for MβCD and 2HβCD, respectively. This can be attributed to the decrease in the polarity inside the cavity, leading to the escape of water molecules and creating more favorable conditions for loading a hydrophobic guest into the cavity. However, it seems that the functionalization of βCD has not been result in a significant change in the number of water molecules in the second inner layer (0.5–0.8nm), as observed in Table 1 when comparing 2HβCD and MβCD to βCD.

**Table 1:** Number of water molecules in different spheres inside the CDs cavity.

Distance Systems	0–0.5 nm	0.5–0.8 nm	0.8–0.9 nm	0.9–1.0 nm	0–1.0 nm (total)
Water + βCD	9.56 (± 0.13)	20.38 (± 0.27)	22.44 (± 0.36)	46.45 (± 0.52)	98.84 (± 0.47)
Water + βCD + cholesterol	0.07 (± 0.02)	11.07 (± 0.30)	20.47 (± 0.42)	46.10 (± 0.57)	77.72 (± 0.45)
Water + MβCD	0.90 (± 0.06)	19.53 (± 0.60)	27.41 (± 0.42)	36.08 (± 0.71)	83.93 (± 0.45)
Water + MβCD + cholesterol	0.10 (± 0.04)	7.63 (± 0.23)	15.14 (± 0.36)	38.25 (± 0.61)	61.14 (± 0.50)
Water + 2HβCD	1.56 (± 0.10)	21.54 (± 0.38)	23.06 (± 0.28)	34.73 (± 0.47)	80.89 (± 0.19)
Water + 2HβCD + cholesterol	0.15 (± 0.08)	5.50 (± 0.68)	13.47 (± 0.21)	35.91 (± 0.83)	55.04 (± 0.63)

All results were obtained from the last 10% of the simulation time.

The binding of cholesterol induces substantial changes in the number of water molecules within the three inner layers across all three carriers. Although noticeable changes occur in the second inner layer for βCD and MβCD, with an approximate 46% and 60% decrease, the presence of cholesterol leads to a much more drastic reduction in water molecules in the second layer for 2HβCD, close to a 75% decrease. In the case of the third inner layer (0.8–0.9 nm), βCD shows a tangible but relatively small decrease of approximately 10% in water molecules, whereas MβCD and 2HβCD exhibit much more significant reductions, with a decrease of 45% and 42% in the number of water molecules, respectively. Using the radial distribution function (RDF), the probability of water molecules being present and its arrangement around CDs was determined, as shown in Figure S7. The findings revealed that when cholesterol binds to CDs, not only does water exit from the center of the cavity, but there was also a significant rearrangement of water molecules up to a radius of approximately 0.9 nm. Overall, it appears that the functionalization of βCD only leads to a change in the number of water molecules in the innermost layer, while cholesterol binding causes a change in all three inner layers and rearranges water molecules up to a radius of 0.9 nm.

### Conformational changes of CDs

The conformational changes in CDs are pivotal for their functional versatility and application potential in various fields, including pharmaceuticals, chemistry, materials science, and biotechnology. These changes directly impact the structure, stability, selectivity, and solubility of CDs, making them valuable tools in molecular encapsulation, recognition, and controlled release applications^57^. Hence, various simulated systems were examined to identify the key conformational changes in CDs, which are summarized in Table 2 and Table S4-S6. The computation methods for determining the area and volume of the cavity were elucidated in the supplementary information (page 11), and these approaches have been well confirmed in other simulation studies^58,59^. One of the major structural changes caused by the functionalization of βCD is the noticeable alteration in the areas of secondary hydroxyl rim (*A*_*SHR*_) and middle rim (*A*_*MID*_) of 2HPβCD and MβCD structures. Specifically, the *A*_*MID*_ value of βCD decreased from 0.83 to 0.70 and 0.78 (nm^2^) for MβCD and 2HPβCD, respectively. These changes were even more pronounced in the *A*_*SHR*_ values, with βCD decreasing from 1.32 to 1.08 and 1.13 (nm^2^) for MβCD and 2HPβCD, respectively. This observation could be attributed to a decrease in the polarity of the cavity, particularly in these two regions, which causes the cavity to have a greater tendency to constrict. This phenomenon is confirmed by examining the volume of the cavity (*V*_*C*_). As shown in Table 2, βCD has the largest volume without a guest, but functionalizing βCD and forming MβCD and 2HPβCD significantly decrease *V*_*C*_. The *V*_*C*_ of βCD decreases from 0.55 to 0.30 and 0.32 (nm^3^) for MβCD and 2HPβCD, respectively. Therefore, it is important to note that functionalizing βCD not only enhances the solubility of the new carrier but also brings about fundamental changes in its configuration. Examining the distances between the rims reveals another aspect, which is that the contraction of the main scaffold of CDs is not solely accompanied by horizontal contraction and a decrease in the areas and the volume of the cavity. The CDs heights (*h*_12_ and *h*_16_) also experience a significant reduction. Specifically, the values of 0.33 (*h*_16_) and 0.22 (*h*_12_) nm in βCD decreased to 0.22 and 0.12 nm in MβCD, and to 0.21 and 0.12 nm in 2HPβCD. Additionally, the circularity (Ω) also undergoes noticeable changes due to the functionalization of βCD, although the degree of freedom of the O1 atoms is lower, and more severe changes for Ω_O2_ and Ω_O6_ are seen. Nevertheless, upon the entry of cholesterol into the cavity and the resulting nonpolar-nonpolar interaction (cholesterol-cavity), the areas (*A*_*PHR*_, *A*_*MID*_, and *A*_*SHR*_) increase in all CDs, with only a partial decrease observed in the *A*_*SHR*_ in βCD. Furthermore, the 2HPβCD and MβCD structures become more circular, leading to a complete increase in Ω_O1_, Ω_O2_, and Ω_O6_ in the presence of cholesterol. Upon cholesterol binding, CDs expand, and the volume of the cavities also increases in all cholesterol-containing systems. The findings related to replicas were also entirely the same. Only in cases where the mechanism of cholesterol binding is such that the oxygen of cholesterol is oriented towards the PHR, an increase in *A*_*PHR*_ is observed due to spatial congestion and the repulsion of bulky rings connected to oxygen. Conversely, when the binding process occurs in the opposite direction (oxygen of cholesterol facing the SHR), the *A*_*SHR*_ remains unchanged, and there is only a minor alteration in APHR. This analysis remains applicable to MβCD as well. For 2HPβCD, because a single mechanism of cholesterol binding was identified, similar structural changes are observed across all systems.

**Table 2:** Conformational parameters describing molecular arrangement of CDs in different simulated systems.

Structural properties Systems	*A*_PHR_ (nm^2^)	*A*_MID_ (nm^2^)	*A*_SHR_ (nm^2^)	Ω_O1_	Ω_O2_	Ω_O6_	*h*_12_ (nm)	*h*_16_ (nm)	*V*_C_ (nm^3^)
Water + βCD	1.10	0.83	1.32	0.99	0.99	0.87	0.22	0.33	0.55
Water + βCD + cholesterol	1.18	0.84	1.28	0.99	0.99	0.88	0.23	0.33	0.57
Water + MβCD	1.05	0.70	1.08	0.90	0.80	0.64	0.12	0.22	0.30
Water + MβCD + cholesterol	1.33	0.84	1.26	0.96	0.98	0.85	0.22	0.34	0.59
Water + 2HβCD	1.22	0.78	1.13	0.98	0.79	0.65	0.12	0.21	0.32
Water + 2HβCD + cholesterol	1.34	0.85	1.21	0.98	0.97	0.83	0.23	0.32	0.58

*A*_PHR_, *A*_MID_, and *A*_SHR_ are the areas of primary hydroxyl rim, middle rim, an secondary hydroxyl rim (the rings that are formed by connecting the O6, O1, and O3 atoms, respectively); *h*_1j_ is the distance between the center of mass of O1 atoms and the center of mass of Oj atoms; Ω_Xi_ is the circularity of rim comprising Oi atoms defined as the ratio of the smallest to the largest distance between any pair of Oi atoms in the rim; CDs height, *h*, is the distance between the centers of mass of the primary and the secondary hydroxyl rims (*h* = *h*_12_ + *h*_16_); Vc is the volume of the cavity for CDs in different simulated systems. All results were obtained from the last 10% of the simulation time.

### Relative shape anisotropy and asphericity parameter

Relative shape anisotropy (RSA), also known as *κ*^*2*^, in polymers pertains to the extent by which the molecular shape of a polymer deviates from that of a perfect sphere or cylinder^60,61^. This characteristic holds significance in the field of drug delivery as it influences the distribution and retention of drug carriers within the body. The RSA parameter plays a crucial role in determining the transport and diffusion of drug carriers through biological barriers like cell membranes and tissue extracellular matrix. Enhancing our understanding of the RSA of drug carriers can lead to improved efficacy in drug delivery and a reduction in potential side effects^62,63^. The principal moments of the radius of gyration (Rg) are typically arranged in the order λ_1_ ≥ λ_2_ ≥ λ_3_, and by summing these principal moments, the square of Rg can be obtained using the equation: ${{\text{R}}}_{{\text{g}}}^{2}={\uplambda }_{1}+{\uplambda }_{2}+ {\uplambda }_{3}$. Consequently, by utilizing the principal moments, *κ*^*2*^ can be calculated as follows^66^:

2 $${\kappa }^{2}=1-3\frac{({\lambda }_{1}{\lambda }_{2}+{\lambda }_{2}{\lambda }_{3}+{\lambda }_{1}{\lambda }_{3})}{{({\lambda }_{1}+{\lambda }_{2}+{\lambda }_{3})}^{2}}$$

*κ*^2^ encompasses a range of values from 0 to 1, where 0 signifies a polymer conformation exhibiting high symmetry, while a value of 1 represents an ideal linear chain configuration. The last descriptor, the asphericity parameter (*b*), measures the deviation of a surface or object from perfect sphericity, quantifying its non-spherical shape and is defined as:

3 $$b={\lambda }_{1}-\frac{1}{2} ({\lambda }_{2}+{\lambda }_{3})$$

**Figure 4:**
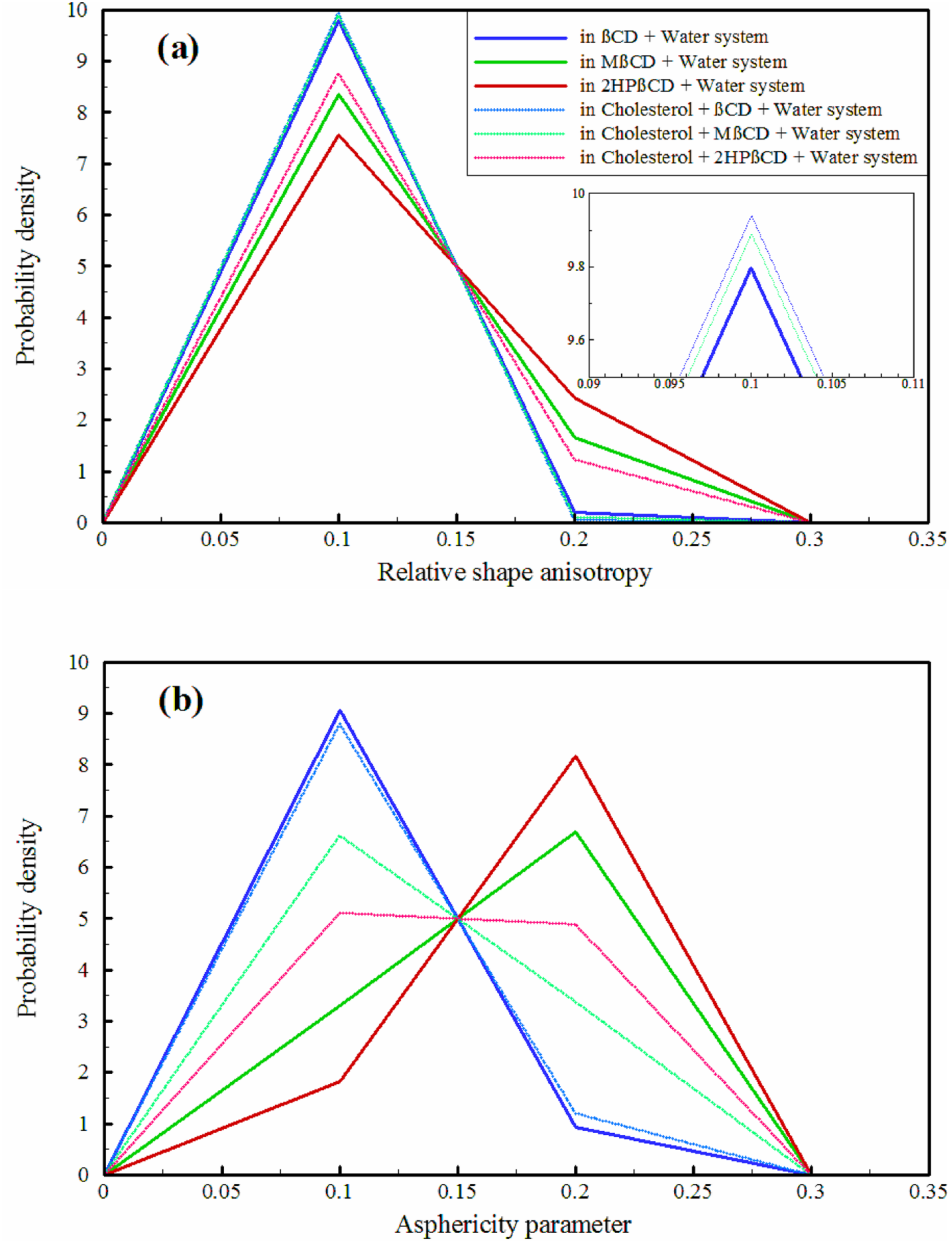
The relative shape anisotropy parameter (**a**), and the asphericity parameter (**b**) of CDs in different simulated systems.

A spherically symmetric object has a value of *b* equal to 0. The examination of the *κ*^2^ and *b* values of CDs were conducted both with and without the presence of cholesterol. The probability density of *κ*^2^ and *b* for CDs were significant in the range of 0.1 (more spherical) to 0.3 (less spherical), as shown in Fig. 4. The results indicate that the probability density of *κ*^*2*^ and *b* for βCD did not significantly change in the presence or absence of cholesterol, and the maximum probability is observed at approximately 0.1, concerning the replicas, the results were entirely identical (Fig. S8). In comparison to βCD and in the absence of cholesterol, a more non-spherical structure is observed for 2HPβCD and MβCD, resulting in a significant decrease in the probability density around 0.1 for *κ*^2^ and the maximum probability around 0.2 for *b*. However, the loading of cholesterol causes both 2HPβCD and MβCD to undergo a substantial transformation into more spherical shapes. This transformation results in a remarkable rise in the maximum probability of *κ*^2^ near 0.1 and a shift in the maximum probability for *b* towards 0.1. As observed, after loading cholesterol, the maximum peak of *κ*^2^ for MβCD was significantly higher compared to 2HPβCD. Additionally, the value of *b* for 2HPβCD still maintains a relatively significant maximum peak at 0.2. Concerning the replicas of MβCD and 2HPβCD, the alterations followed a similar pattern. Nonetheless, there was a slight difference in the probability density around these discretized values indicated, as seen in Figs. S9 and S10.

### Thermodynamic properties

The solubility and stability of a compound in a solution are heavily influenced by the energies involved in the interaction between the solute and solvent. These energies are mainly influenced by Coulombic forces, vdW forces, and hydrogen bonding interactions. The strength and directionality of these interactions are influenced by the chemical properties and geometry of both the solute and solvent molecules. Comprehending these interactions is crucial when it comes to creating innovative carriers, enhancing the circumstances for reactions, and developing new materials^19,64^.

#### Solvation and binding free energy

**Figure 5:**
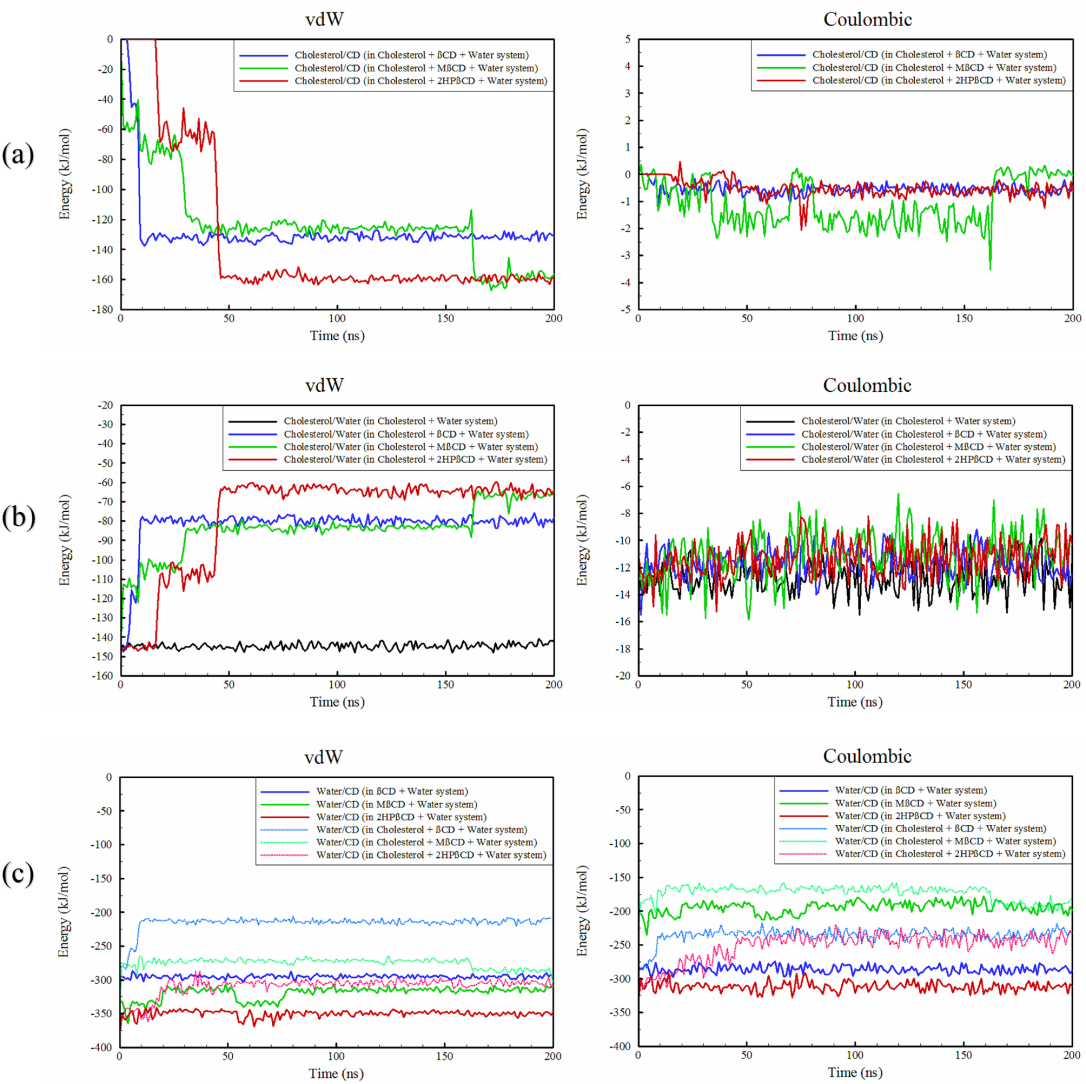
Time evaluation of interaction energies between different components at the simulated systems.

The BAR method was employed to determine the solvation free energy (ΔG_solv_) and binding free energy (ΔG_bind_) of cholesterol to CDs, as described above. The ΔG_solv_ of cholesterol in water was found to be 11.18 kJ/mol, as expected due to the hydrophobic nature of cholesterol. Furthermore, the ΔG_bind_ values of cholesterol to βCD, MβCD, and 2HPβCD were -77.49, -75.55, and -68.94 kJ/mol, respectively. It is evident that the binding of cholesterol to all three CDs is spontaneous and energetically favorable. Among these, the most exothermic processes, in order, are related to the binding of cholesterol to βCD, MβCD, and 2HPβCD. Additional details regarding the forces involved in the binding and solvation were revealed by examining various types of interaction energies in simulated systems. Figure 5a demonstrates that vdW interactions play a dominant and decisive role in the binding of cholesterol to CDs, while the contribution of Coulombic interactions is negligible. Simultaneously, the examination of cholesterol/water interactions shows that (Fig. 5b) although the contribution of Coulombic interactions is somewhat significant (even after binding to CDs), vdW interactions still dominate and play a driving role in the cholesterol/CDs binding process throughout the simulation time. Through the comparison of systems comprising solely of CDs without cholesterol, it becomes apparent that the functionalization of βCD and the creation of 2HPβCD and MβCD notably enhance the vdW interactions, causing them to become more negative, as shown in Fig. 5c. This enhancement leads to an increased solubility in both new carriers, namely 2HPβCD and MβCD. However, it is observed that the Coulombic interactions are only strengthened in 2HPβCD, whereas in MβCD, a repulsive effect takes place, resulting in a reduction of approximately 30% in the Coulombic interaction energy between water/MβCD as compared to water/βCD. In the presence of cholesterol, a repulsive interaction occurs between water/CDs. The strongest vdW repulsion is observed in the interaction between water/βCD, which in turn leads to a significant decrease in the solubility of cholesterol/βCD complex compared to cholesterol/2HPβCD complex (Fig. S11). Compared to βCD, MβCD exhibits a notable Coulombic repulsion due to its functionalization, as well as vdW and Coulombic repulsions induced by the binding of cholesterol, which leads to cholesterol/MβCD having approximately the same solubility as cholesterol/βCD until around 160 ns. Afterward, between 160 and 200 ns, a noticeable reduction in both vdW and Coulombic interaction energies occurs between MβCD and water. This change coincides with the alteration of the configuration and the vertical binding of cholesterol within the MβCD cavity. As a result, the solubility of the cholesterol/MβCD becomes slightly higher compared to the cholesterol/βCD. The analysis of the interaction energies among different components in all the simulated systems (Tables S7–S9) reveals that the thermodynamic behavior of systems containing cholesterol and each CD remains consistent, regardless of the initial position of cholesterol relative to the CD. Moreover, once cholesterol binds to the cavity of the CD, it maintains complete stability from a thermodynamic viewpoint.

#### Hydrogen bonds

The importance of hydrogen bonding is crucial in "host–guest" systems, as it plays a key role in selective binding, stability, shape recognition, control of properties, reversibility, and even influences the pharmacokinetics of drugs^65,66^. Therefore, the evaluation of the capability to form hydrogen bonds between various components in simulation systems was conducted, and the results are presented in Table 3 and Tables S11–S13. As seen, in the absence of cholesterol, βCD and 2HPβCD exhibit higher number of hydrogen bonds with water compared to MβCD. Specifically, βCD and 2HPβCD form 19.41 and 19.97 hydrogen bonds with water, respectively, while MβCD forms 11.40. The solubility of CDs is mainly related to their molecular structure. For example, a completely cyclic hydrogen bond is formed in the molecule of the βCD, which makes the molecule quite rigid, leading to its decrease in the solubility of water. Removal of this network by ionization of the hydroxyl groups leads to a greatly increased solubility and removal of aggregation. Through derivative formation, it is possible to enhance their solubility and complexation capacity^17,67^. In this context, the modification of βCD resulted in changes in the number of internal hydrogen bonds (CD/CD). Specifically, the hydrogen bonding between MβCD/MβCD (2.14) was significantly reduced by approximately 64%, compared to the original βCD value of 5.94. Similarly, the hydrogen bonding between 2HβCD/2HβCD (4.08) decreased by about 32%. These outcomes imply that the decrease in hydrogen bonding CD/CD could potentially result in heightened flexibility, as was indicated by the pronounced reduction in the volumes of the cavities in MβCD and 2HβCD structures when compared to the rigid βCD structure. This reduction in internal hydrogen bonding could also contribute to an improved solubility of the MβCD and 2HβCD structures. However, it is notable that the lifetime of the hydrogen bonds in 2HPβCD/2HPβCD was considerably shorter, lasting only 19.57 ps. The dipole moment of βCD was 4.29 Debye. Due to functionalization and forming 2HPβCD and MβCD, the dipole moment increased to 5.47 and 6 Debye, respectively. Nonetheless, this polarization effect is prominent exclusively in 2HPβCD, where there was a substantial increase in the acceptor (Table S14). On the other hand, MβCD experienced a significant decrease in the number of donors while maintaining a constant number of acceptors. Consequently, the increasing in dipole moment is accompanied solely by an augmentation in hydrogen bonding within 2HPβCD, while MβCD exhibits a notable decrease in hydrogen bonding with water. When cholesterol is bonded, both βCD and 2HPβCD still possess the highest number of hydrogen bonds with water, approximately 15 for both. The number of hydrogen bonds formed between cholesterol/CDs exhibited negligible changes, indicating that hydrogen bonding does not play a prominent role to the binding process between cholesterol and CDs. Furthermore, because only the hydroxyl group present in cholesterol is capable of forming hydrogen bonds with water, and considering that this part of cholesterol is equally accessible to water molecules in all three carriers, the number of cholesterol/water hydrogen bonds was the same in all systems. The most interesting result pertains to the number of hydrogen bonds between CD/CD, which demonstrated a significant increase by the cholesterol binding in all carriers, with the most significant increase being related to 2HPβCD.

**Table 3:** Average number of different hydrogen bonds and life-time (ps) in the simulated systems.

H-bond System	Between water and cholesterol (life-time)	Between water and CD (life-time)	Between cholesterol and CD (life-time)	Between CD and CD (life-time)
Water + βCD	–	19.41 (10.69)	–	5.94 (29.02)
Water + βCD + cholesterol	1.02 (10.75)	15.04 (10.75)	0.002 (10.00)	7.51 (37.91)
Water + MβCD	–	11.40 (11.20)	–	2.14 (29.05)
Water + MβCD + cholesterol	1.06 (10.90)	10.38 (11.58)	0.000 (0.00)	4.29 (44.97)
Water + 2HβCD	–	19.97 (11.13)	–	4.08 (± 19.57)
Water + 2HβCD + cholesterol	0.98 (10.76)	15.42 (10.90)	0.005 (10.00)	8.46 (34.97)
Water + cholesterol	1.02 (10.58)	–	–	–

All results were obtained from the last 10% of the simulation time.

#### The free energy landscape (FEL)

**Figure 6:**
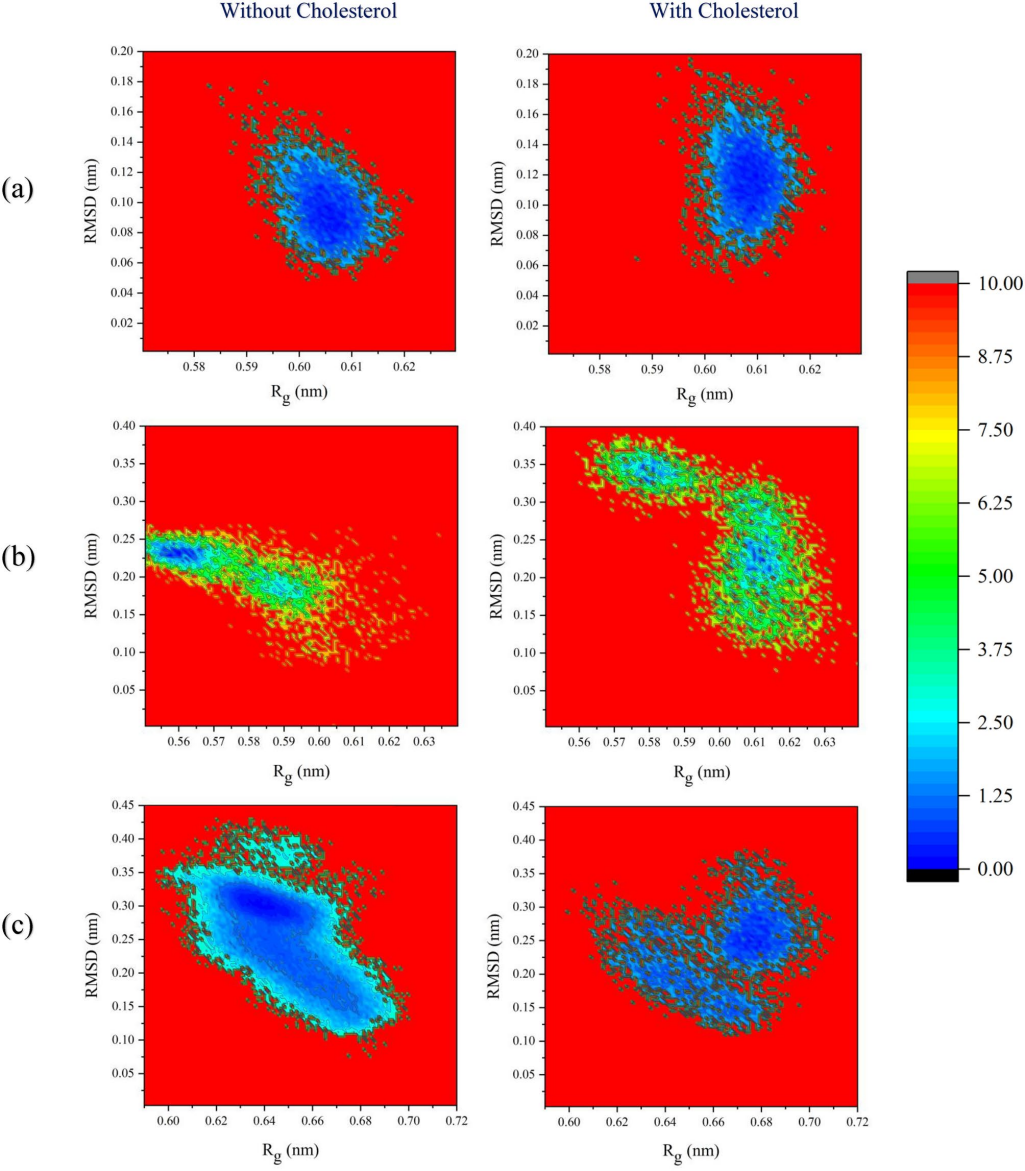
The free energy landscapes as a function of the root-mean-square deviation (RMSD) and the radius of gyration (Rg) for βCD (**a**), MβCD (**b**), and 2HPβCD (**c**).

The FEL is a concept used in physics, chemistry, and biochemistry to describe all possible conformations of the spatial positions of interacting molecules in a system. Understanding these landscapes enables us to understand the relationships between structure, dynamics, stability, and functional behavior of molecules such as polymers and proteins. It can also help identify stable states or meta-stable intermediates, and elucidate underlying transition states^68–71^. Therefore, in this study, considering two important collective variables, radius of gyration (Rg) and root mean square deviation (RMSD), which describe the fundamental motions and coordinates of the CDs, the FEL was investigated as shown in Fig. 6. The minimized structures were considered as the reference for RMSD, and no bias potential was applied in this process. In the absence of cholesterol, βCD exhibits a dominant structure characterized by a Rg of 0.60 nm and a RMSD of 0.1 nm, which represents its most stable conformation. The binding of cholesterol causes only slight alterations in the structure, where the more stable conformation still maintains an Rg of 0.61 nm and an RMSD of 0.12 nm. On the other hand, in the case of MβCD, in the absence of cholesterol, a more stable structure with an Rg of 0.56 nm and an RMSD of 0.24 nm is observed, although there is a tendency for structures with slightly larger Rg and slightly lower RMSD values to form. The energy barrier for this transformation is estimated to be around 3–4 kJ/mol. Comparatively, MβCD exhibits a smaller Rg than βCD, indicating reduced cavity polarity and volume, as discussed in “Conformational changes of CDs”. However, upon cholesterol binding to MβCD, there is an increased likelihood of observing structures with significantly larger Rg (up to approximately 0.63 nm) and significantly larger RMSD values (up to approximately 0.34 nm). The energy barrier for transitioning into these structures appears to be higher. An interesting characteristic of MβCD is the wide range of RMSD values observed, both in the absence and presence of cholesterol, indicating greater flexibility compared to βCD. It can be inferred that MβCD can thermodynamically adopt two stable structures. In the absence of cholesterol, 2HPβCD exhibits a dominant structure with an Rg of approximately 0.64 nm and an RMSD of 0.3 nm. Upon cholesterol binding, the highest likelihood is observed for structures with an Rg around 0.68 and an RMSD between 0.25 nm and 0.3 nm, indicating a thermodynamic preference. Similar to MβCD, 2HPβCD also exhibits a wide range of structures with different RMSD values, indicating increased flexibility compared to βCD.

## Conclusion

This investigation employed a set of molecular dynamics simulations to elucidate the dynamics and thermodynamics effects arising from the interaction between cholesterol and several widely recognized cyclodextrins, specifically β-Cyclodextrin (βCD), 2-Hydroxypropyl-βCD (2HPβCD), and Heptakis(2,6-di-*O*-methyl)-βCD (MβCD). The results showed that cholesterol rapidly binds to MβCD, followed by βCD and 2HPβCD with longer time intervals. Through the examination of all simulated systems, it was found that the oxygen atom of cholesterol is positioned outside the CD cavities. This orientation is due to the hydrophilic nature of oxygen, which leads to its more favorable interaction with water. Furthermore, its preferred orientation in βCD and MβCD is inclined towards both the primary and secondary hydroxyl rims, while in 2HPβCD it is oriented solely towards the primary hydroxyl rim. Cholesterol binding caused significant changes in the number of water molecules across all three CDs and rearranged water molecules up to a radius of 0.9 nm. The functionalization of βCD also led to alterations in the secondary hydroxyl rim (*A*_*SHR*_) and middle rim (*A*_*MID*_) areas, resulting in a decrease in cavity volume and CD heights. Upon cholesterol binding, the CDs expanded, and the cavity volume increased. The circularity of the CDs increased, indicating a more spherical shape. The loading of cholesterol induced structural transformations in 2HPβCD and MβCD, making them more spherical compared to βCD. The solvation free energy (ΔG_solv_) of cholesterol in water was determined to be 11.18 kJ/mol, consistent with its hydrophobic nature. The binding free energies (ΔG_bind_) of cholesterol to the three CDs were found to be −77.49, kJ/mol for βCD, −75.55 kJ/mol for MβCD, and −68.94 kJ/mol for 2HPβCD, indicating spontaneous and energetically favorable binding processes. Through detailed analysis of interaction energies, it was observed that vdW interactions played a dominant role in the binding of cholesterol to CDs, while Coulombic interactions had negligible contribution. The functionalization of βCD and the creation of 2HPβCD and MβCD enhanced the vdW interactions, leading to increased solubility in the new carriers. However, Coulombic interactions were only strengthened in 2HPβCD, while a repulsive effect occurred in MβCD, resulting in a reduction of the Coulombic interaction energy between water/MβCD. Hydrogen bonding was found to play a minor role in the binding process between cholesterol and CDs, as the number of cholesterol/water hydrogen bonds remained almost constant across all systems. However, the binding of cholesterol caused a significant increase in the number of hydrogen bonds between CD/CD, particularly in 2HPβCD. The examination of free energy landscapes (FEL) revealed that when cholesterol binds, it induces small changes in the shape of βCD. On the other hand, 2HPβCD and MβCD displayed a thermodynamic preference for structures with a slightly larger radius of gyration (Rg) and root-mean-square deviation (RMSD) values. 2HPβCD and MβCD demonstrated higher flexibility in comparison to βCD, and it had the ability to adopt two stable structures.

### Supplementary Information

Supplementary Information.

## Data Availability

The datasets used and/or analyzed during the current study are available from the corresponding author on reasonable request.
