# Supplementary material for: Exploring the role of cyclodextrins as a cholesterol scavenger: a molecular dynamics investigation of conformational changes and thermodynamics
Source: Sci Rep. 2023 Dec 8;13:21765. doi: 10.1038/s41598-023-49217-8 (PMC10709460; doi:10.1038/s41598-023-49217-8)
Supplement: Supplementary file 1 — Supplementary Information. [file 41598_2023_49217_MOESM1_ESM.pdf]

Supplementary information: Exploring the Role of Cyclodextrins as a Cholesterol Scavenger: A Molecular Dynamics Investigation of Conformational Changes and Thermodynamics

Mokhtar Ganjali Koli[a,b,], Federico Fogolari[c]*

[a]*Department of Chemistry, University of Kurdistan, Sanandaj, Iran*

[b]*Computational Chemistry Laboratory, Kask Afrand Exire Ltd., Sanandaj, Iran*

[c]*Dipartimento di Scienze Matematiche, Informatiche e Fisiche (DMIF), University of Udine, Via delle Scienze 206, 33100 Udine, Italy.*

*** Corresponding Author:**

Email: m.ganjalikoli1360@gmail.com

Contents

Figure S1: Molecular structure and the numbering in Cholesterol (a), and main skeleton of βCD derivatives (b)……………………………………………………………………………..….**3**

Figure S2: Varied initial configurations of Cholesterol placement around CDs in four replicas….**4**

Details of Free energy computations…………………………………………………………**5**

Figure S3: Final configuration of Cholesterol/βCD systems………………………………….….**6**

Figure S4: Final configuration of Cholesterol/MβCD systems……………………………….….**7**

Figure S5: Final configuration of Cholesterol/2HPβCD systems…………………………….….**8**

Figure S6: Time evaluation the distance between Cholesterol and the center of CD molecules in different replicas……………………………………………………………………………..**9**

Table S1-S3: Number of water molecules in different spheres inside the βCD, MβCD, 2HPβCD cavity……………………………………………………………………..………………**10**

Figure S7: Radial distribution function (RDF) of water around CDs in different simulated systems……………………………………………………………..…………….…………**11**

Table S4-S6: Conformational Parameters Describing Molecular Arrangement of βCD, MβCD, 2HPβCD in different simulated systems………………….……………………..………..**12**

Calculation methods for area and volume of cavity…….……..…………………..…….**13**

Figure S8: The relative shape anisotropy parameter (a), and the Asphericity parameter (b) of βCD in different simulated systems…………………..…………………………………..…….**14**

Figure S9: The relative shape anisotropy parameter (a), and the Asphericity parameter (b) of MβCD in different simulated systems…………………..………………………………..….**15**

Figure S10: The relative shape anisotropy parameter (a), and the Asphericity parameter (b) of 2HPβCD in different simulated systems…………………..……………………..………**16**

Figure S11: The total interaction energy between water/CDs in different simulated systems........**17**

Table S7-S9: Analyzing of energies in different simulated systems that containing βCD, MβCD, and 2HPβCD…….……………………..……………………………………………………**18**

Table S10-S12: Average number of different hydrogen bonds in the βCD, MβCD, and 2HPβCD simulated systems……………………………………………………………….…………**19**

Table S13: The number of acceptors and donors involved in hydrogen bonding in this study……………………………………………………………………………….………..**20**

(a)

(b)

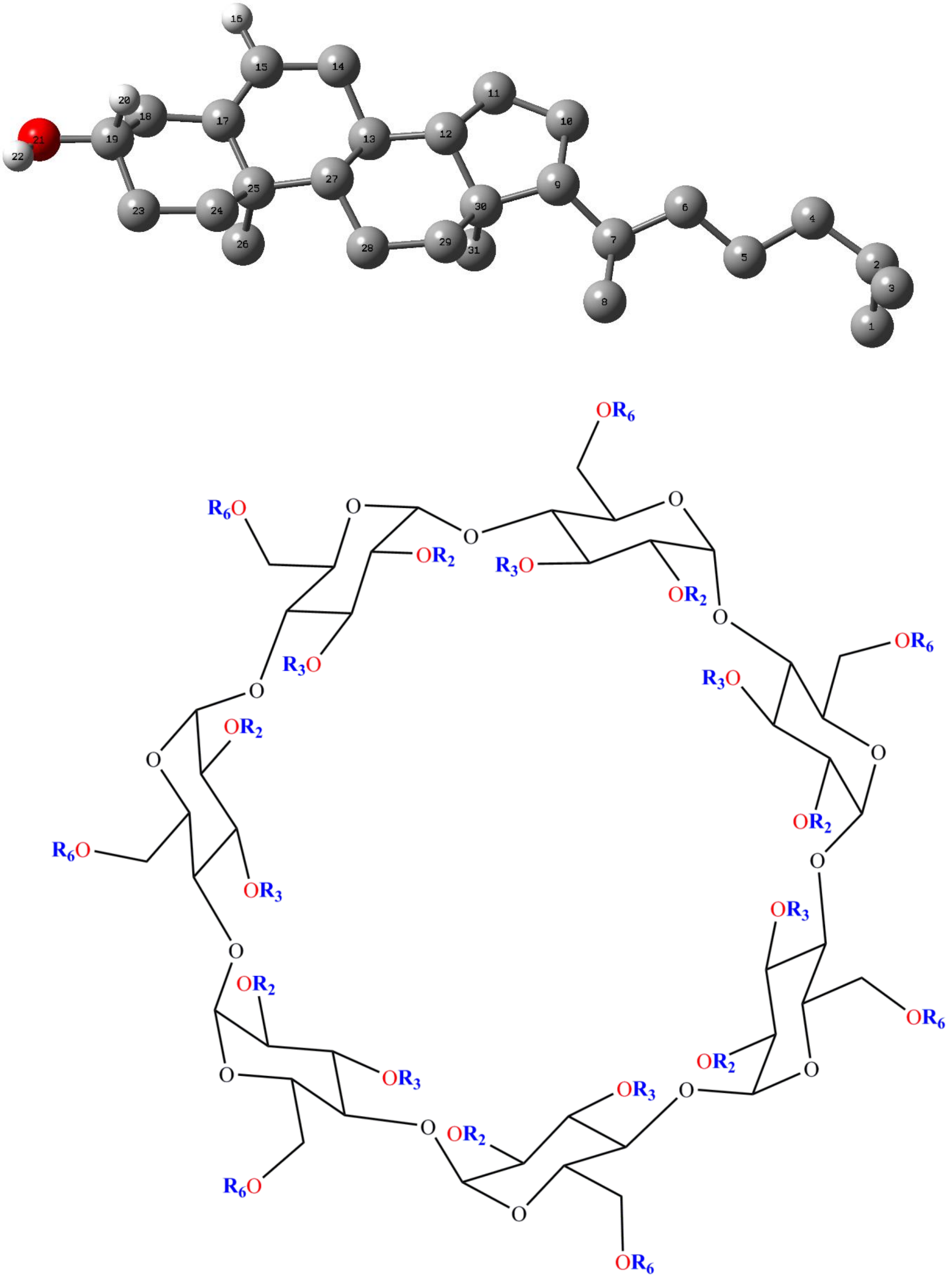

Figure S1: Molecular structure and the numbering in Cholesterol (a), and main skeleton of βCD derivatives (b)

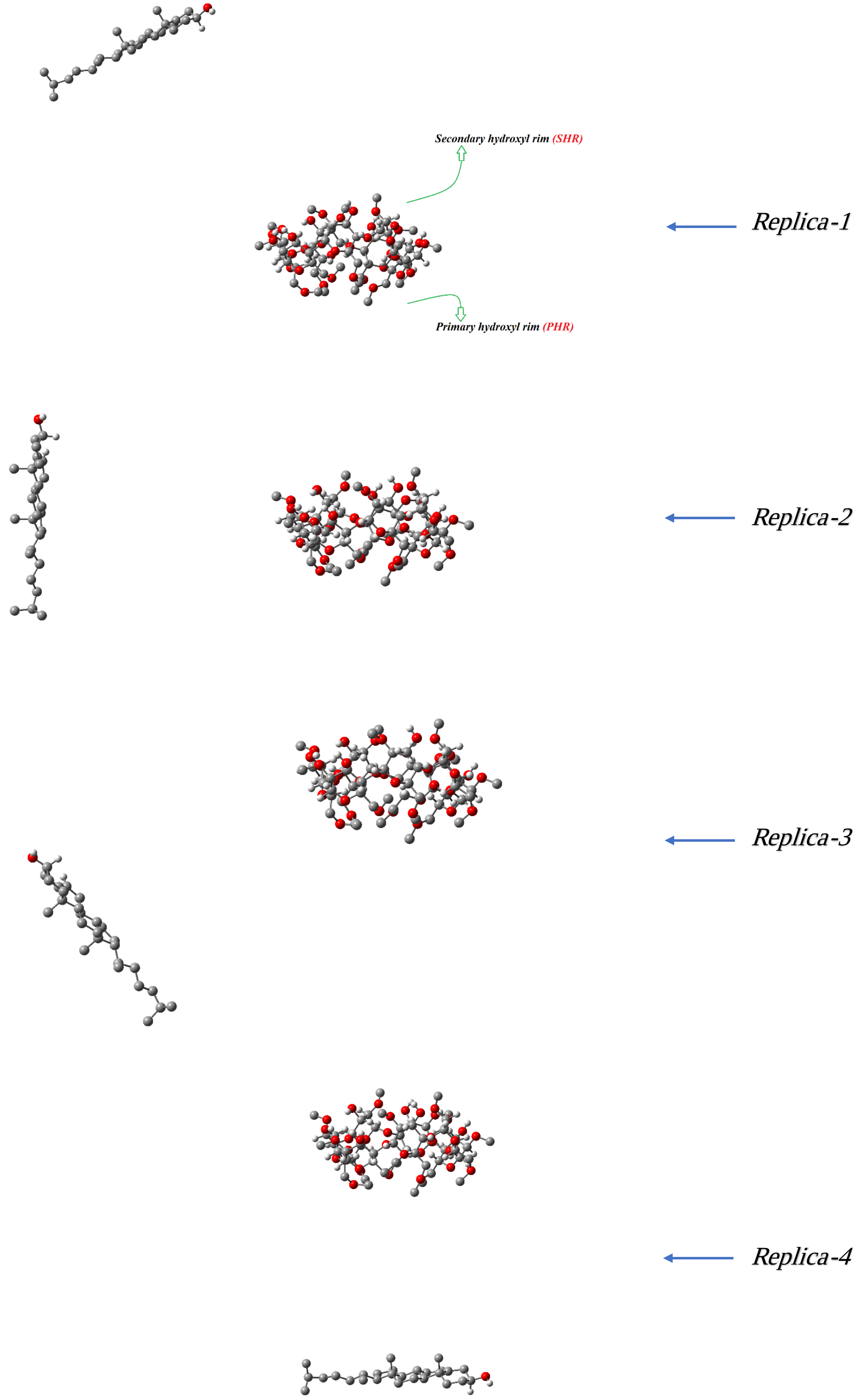

Figure S2: Varied initial configurations of Cholesterol placement around CDs in four replicas.

Details of Free energy computations

Here, two sets of energy calculations were performed.

ΔG₁: Decoupling of Cholesterol interactions in Water:

The final configuration obtained from a 200 ns simulation of a system comprising water and cholesterol was used as the initial configuration for calculating the solvation free energy. The decoupling of cholesterol from the surrounding environment (solely water) was achieved through 25 λ values ranging from 0 to 1. After the simulation, a value of 11.18 kJ/mol was obtained for the solvation free energy of cholesterol.

ΔG_2: Decoupling of Cholesterol interactions in the presence of Water/Cyclodextrins:

In this study, the initial configurations for calculating free energy were obtained from the final configurations of 200 ns simulations for each water/cholesterol/cyclodextrin system. Cholesterol was fully loaded into the cyclodextrin cavities during these simulations. The decoupling of cholesterol from the environment (water/cyclodextrins) was achieved using 25 λ values ranging from 0 to 1. The resulting free energy values for βCD, MβCD, and 2HPβCD were -66.31, -64.37, and -57.76 kJ/mol, respectively.

Following the thermodynamic cycle detailed by Mobley et al. (reference 40), the binding free energy of cholesterol to cyclodextrins was determined as follows:

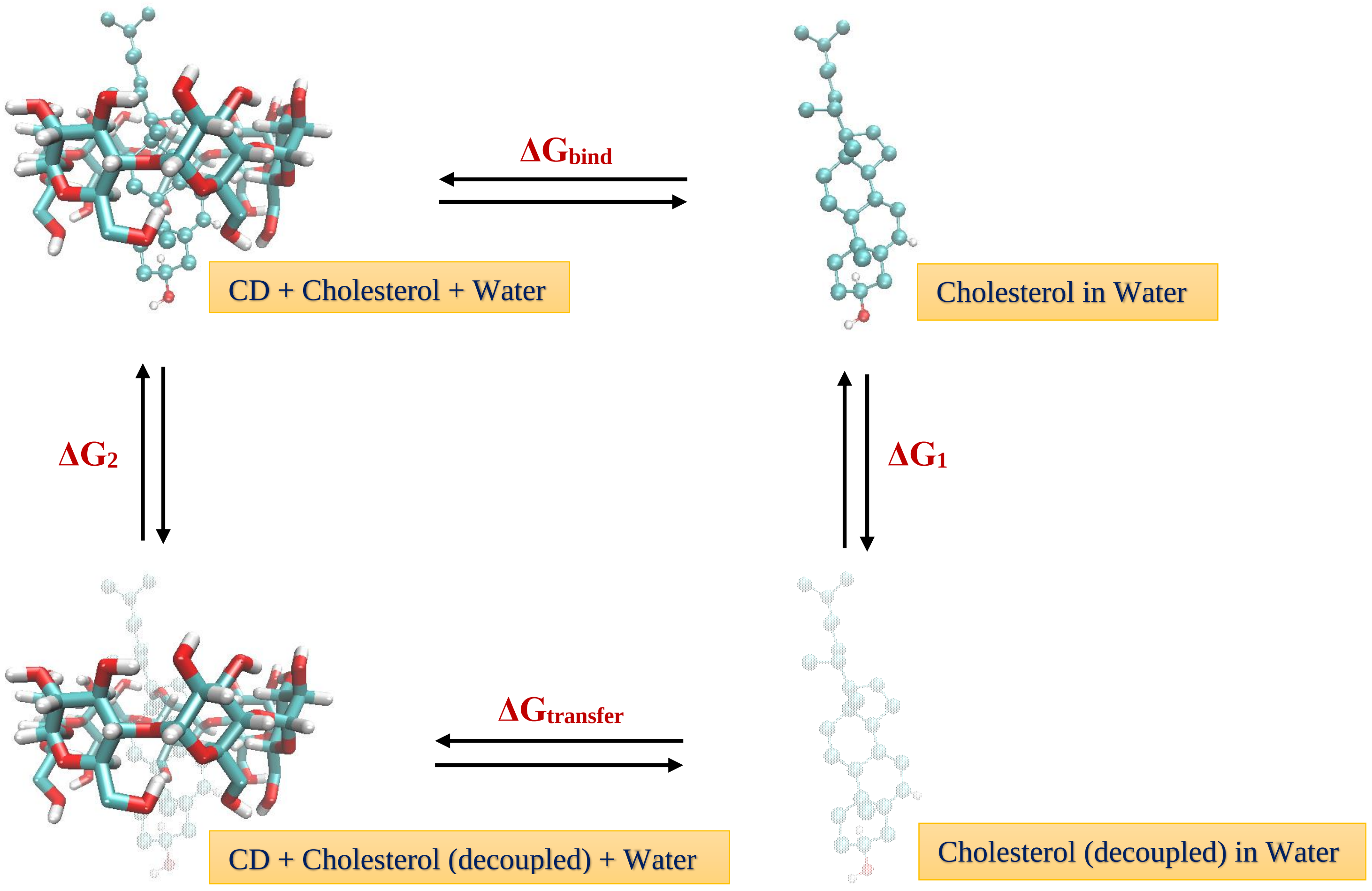

In the above schematic, ΔG_1 is $\Delta G_{solvation}$ of *Cholesterol* in water and ΔG_2 is $\Delta G_{complexation}$ of *CD/Cholesterol*, and $\Delta G_{transfer} = 0$. Considering the following equations, the binding free energy could be calculated:

$$\Delta G_{bind} = \Delta G_{complexation} + \Delta G_{desolvation}$$
$$\Delta G_{solvation} = -\Delta G_{desolvation}$$
$$\Delta G_{bind} = \Delta G_{complexation} - \Delta G_{solvation}$$

In both the complex (ΔG_2) and solvent (ΔG_1) phases of the thermodynamic cycle, the Coulombic interactions of cholesterol were completely annihilated before decoupling the vdW interactions. This annihilation of Coulombic interactions was performed during the first twelve λ. Subsequently, in the next thirteen λ, the vdW interactions were decoupled.

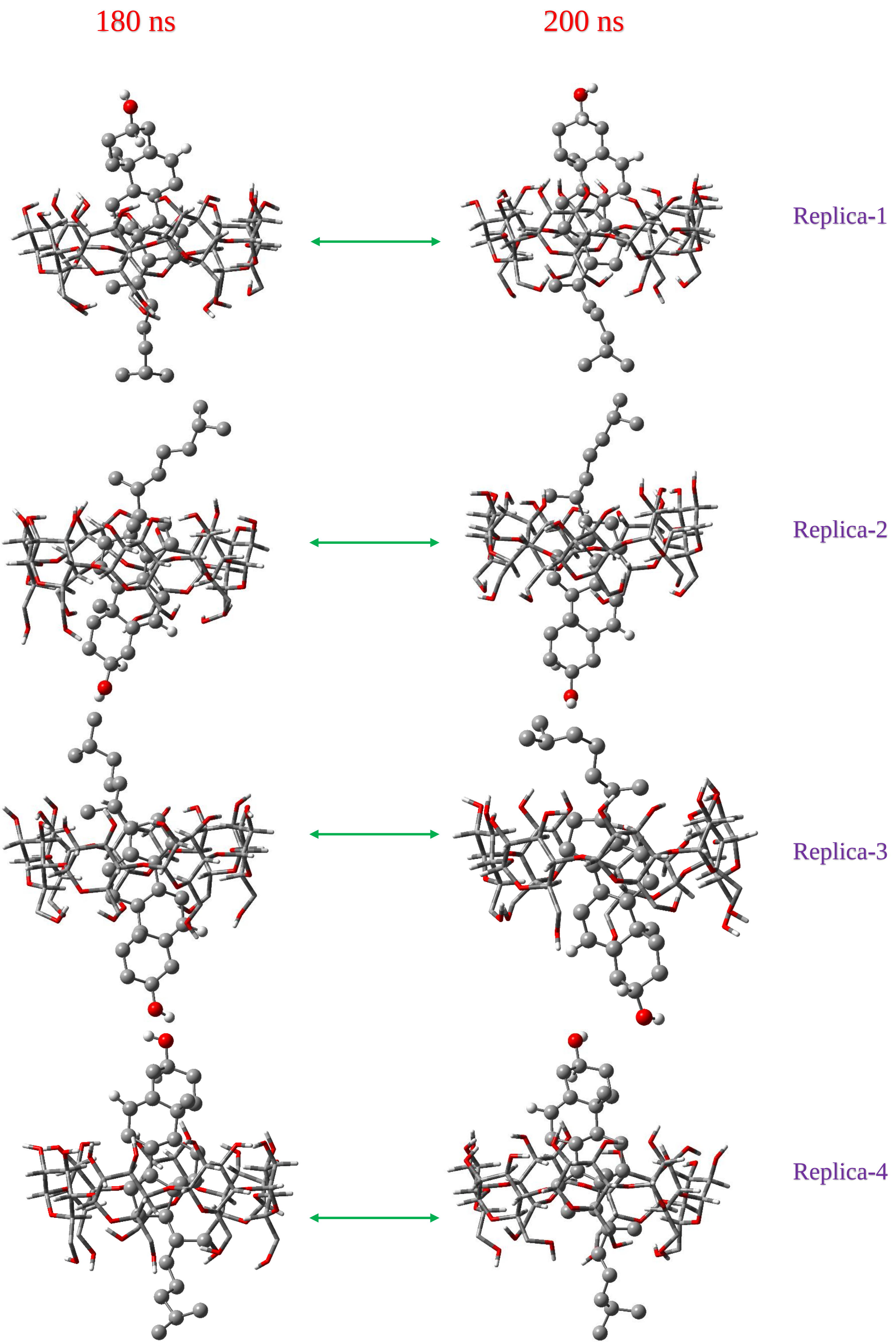

Figure S3: Final configuration of Cholesterol/βCD systems.

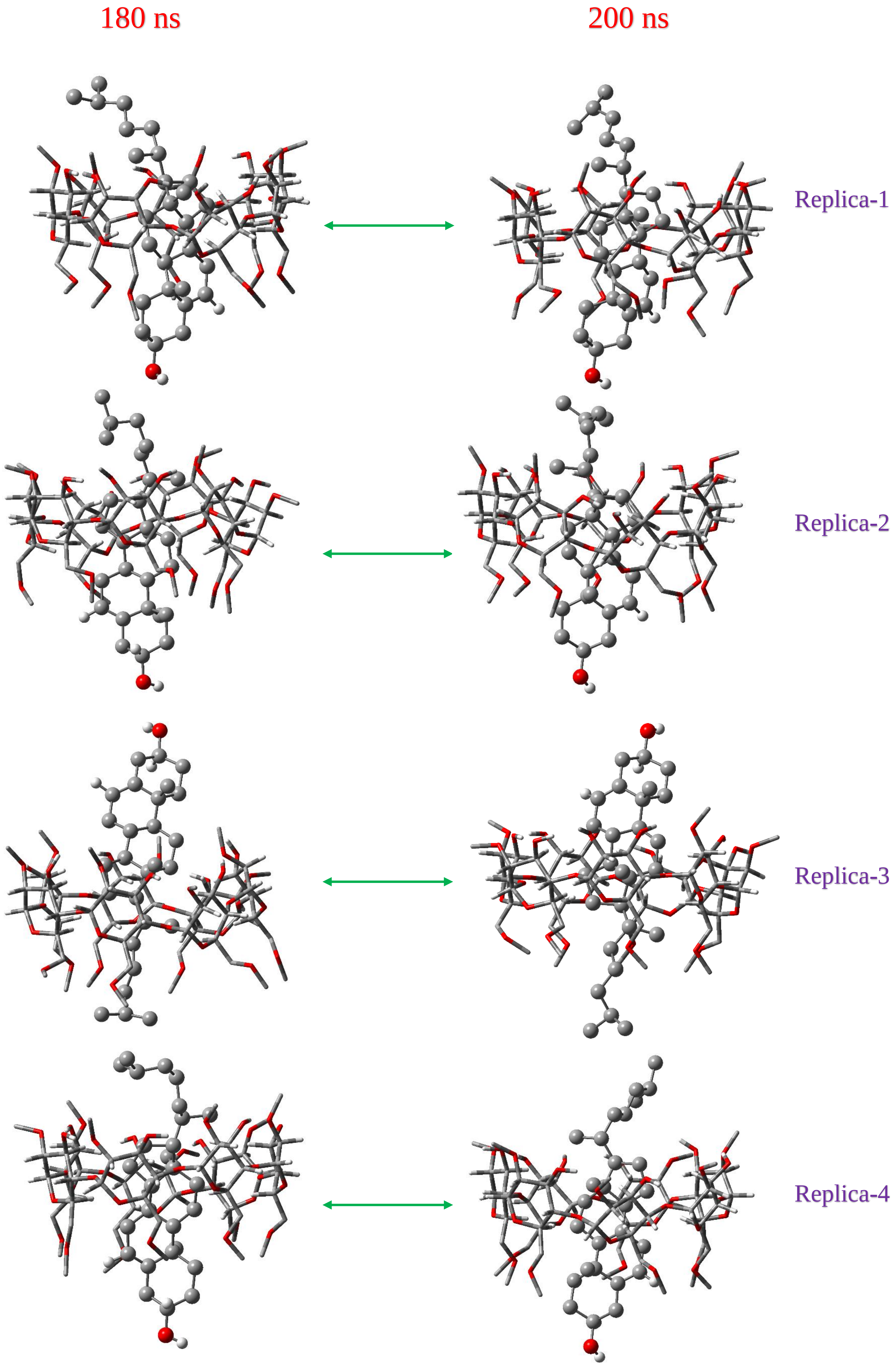

Figure S4: Final configuration of Cholesterol/MβCD systems.

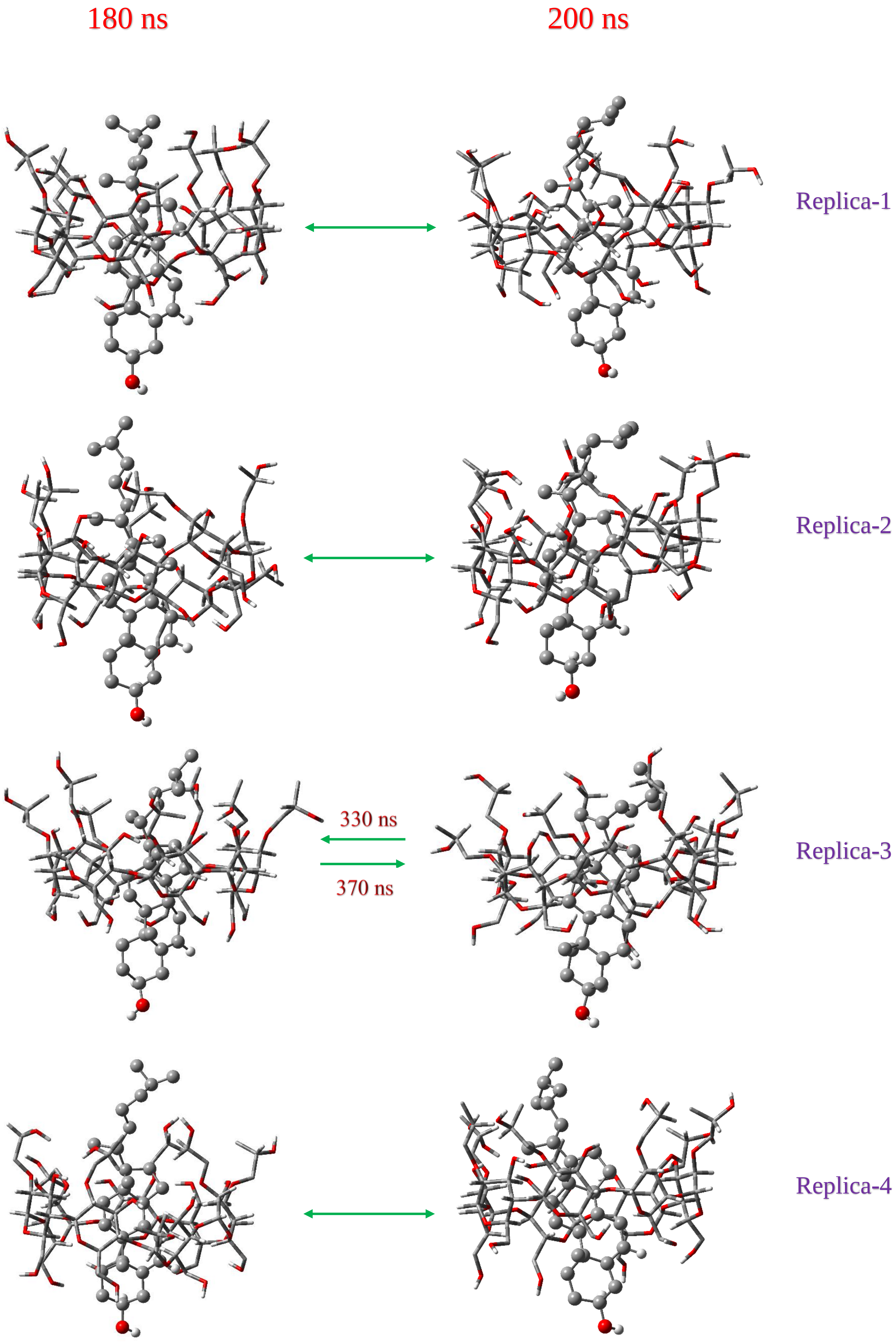

Figure S5: Final configuration of Cholesterol/2HPβCD systems.

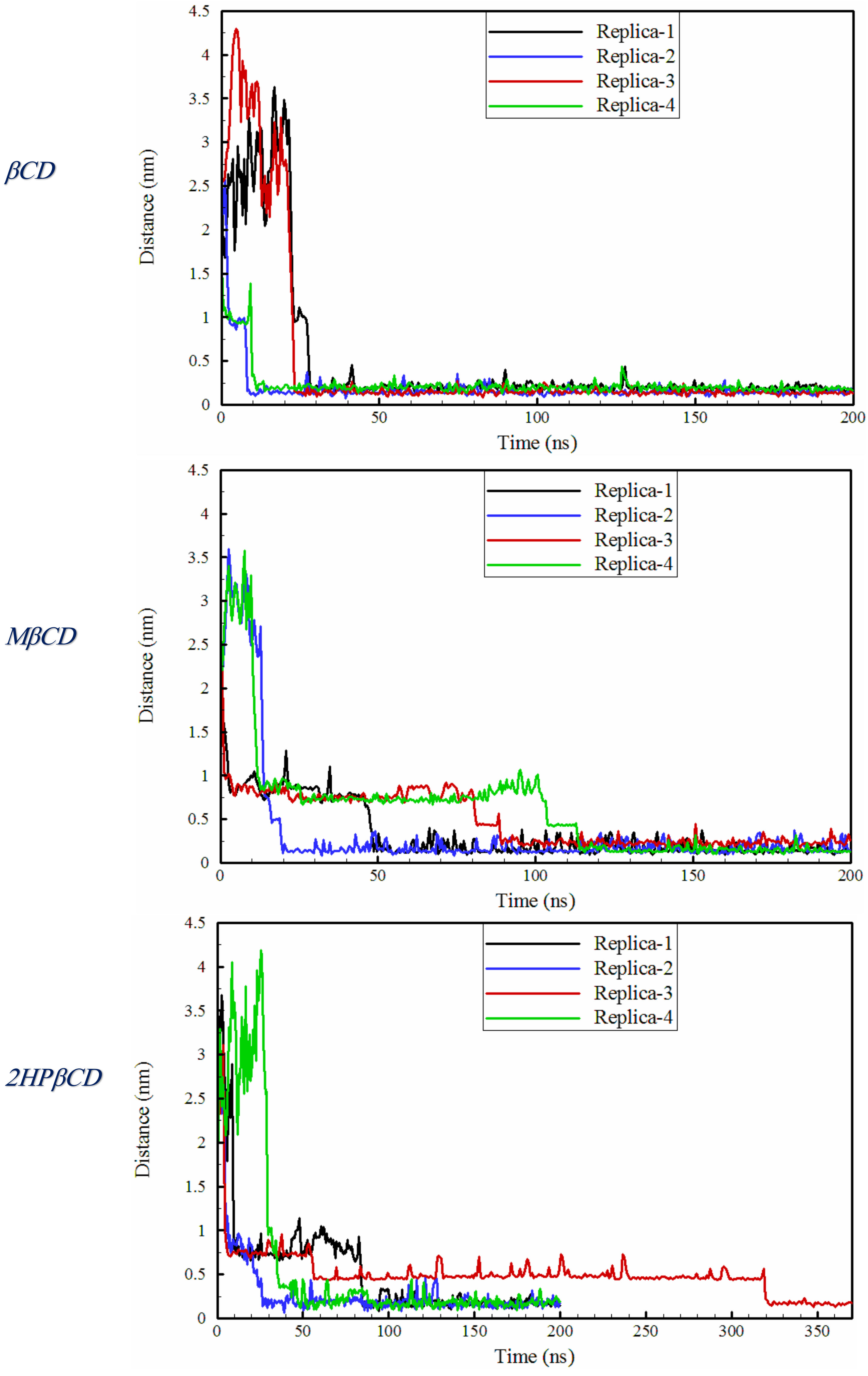

Figure S6: Time evaluation the distance between Cholesterol and the center of CD molecules in different replicas.

Table S1: Number of water molecules in different spheres inside the βCD cavity[a].

Distance / Systems	0-0.5 nm	0.5-0.8 nm	0.8-0.9 nm	0.9-1.0 nm	0-1.0 nm (Total)
**Replica-1**	**0.05 (±0.02)**	**11.00 (±0.28)**	**20.18 (±0.41)**	**45.81 (±0.47)**	**77.25 (±0.40)**
**Replica-2**	**0.06 (±0.02)**	**11.14 (±0.30)**	**20.26 (±0.33)**	**46.19 (±0.44)**	**77.65 (±0.56)**
**Replica-3**	**0.06 (±0.02)**	**11.24 (±0.31)**	**20.37 (±0.38)**	**46.22 (±0.54)**	**77.90 (±0.42)**
**Replica-4**	**0.04 (±0.02)**	**11.03 (±0.26)**	**20.18 (±0.37)**	**46.02 (±0.42)**	**77.27 (±0.48)**

[a]All results were obtained from the last 10 % of the simulation time.

Table S2: Number of water molecules in different spheres inside the MβCD cavity[a].

Distance / Systems	0-0.5 nm	0.5-0.8 nm	0.8-0.9 nm	0.9-1.0 nm	0-1.0 nm (Total)
**Replica-1**	**0.08 (± 0.03)**	**7.94 (± 0.23)**	**15.25 (± 0.39)**	**38.50 (± 0.43)**	**61.78 (± 0.51)**
**Replica-2**	**0.10 (± 0.07)**	**9.06 (± 0.90)**	**15.14 (± 0.35)**	**36.54 (± 0.46)**	**61.11 (± 0.47)**
**Replica-3**	**0.06 (± 0.01)**	**8.48 (± 0.21)**	**15.58 (± 0.37)**	**36.93 (± 0.47)**	**61.05 (± 0.51)**
**Replica-4**	**0.06 (± 0.03)**	**7.78 (± 0.26)**	**15.21 (± 0.38)**	**38.35 (± 0.41)**	**61.41 (± 0.51)**

[a]All results were obtained from the last 10 % of the simulation time.

Table S3: Number of water molecules in different spheres inside the 2HPβCD cavity[a].

Distance / Systems	0-0.5 nm	0.5-0.8 nm	0.8-0.9 nm	0.9-1.0 nm	0-1.0 nm (Total)
**Replica-1**	**0.12 (± 0.06)**	**5.48 (± 0.61)**	**13.35 (± 0.29)**	**35.78 (± 0.53)**	**54.73 (± 0.71)**
**Replica-2**	**0.11 (± 0.07)**	**5.55 (± 0.65)**	**13.39 (± 0.38)**	**36.02 (± 0.50)**	**55.08 (± 0.59)**
**Replica-3**	**0.12 (± 0.07)**	**5.61 (± 0.64)**	**13.29 (± 0.29)**	**36.01 (± 0.63)**	**55.05 (± 0.56)**
**Replica-4**	**0.13 (± 0.08)**	**5.95 (± 0.69)**	**13.71 (± 0.36)**	**36.03 (± 0.56)**	**55.82 (± 0.58)**

[a]All results were obtained from the last 10 % of the simulation time.

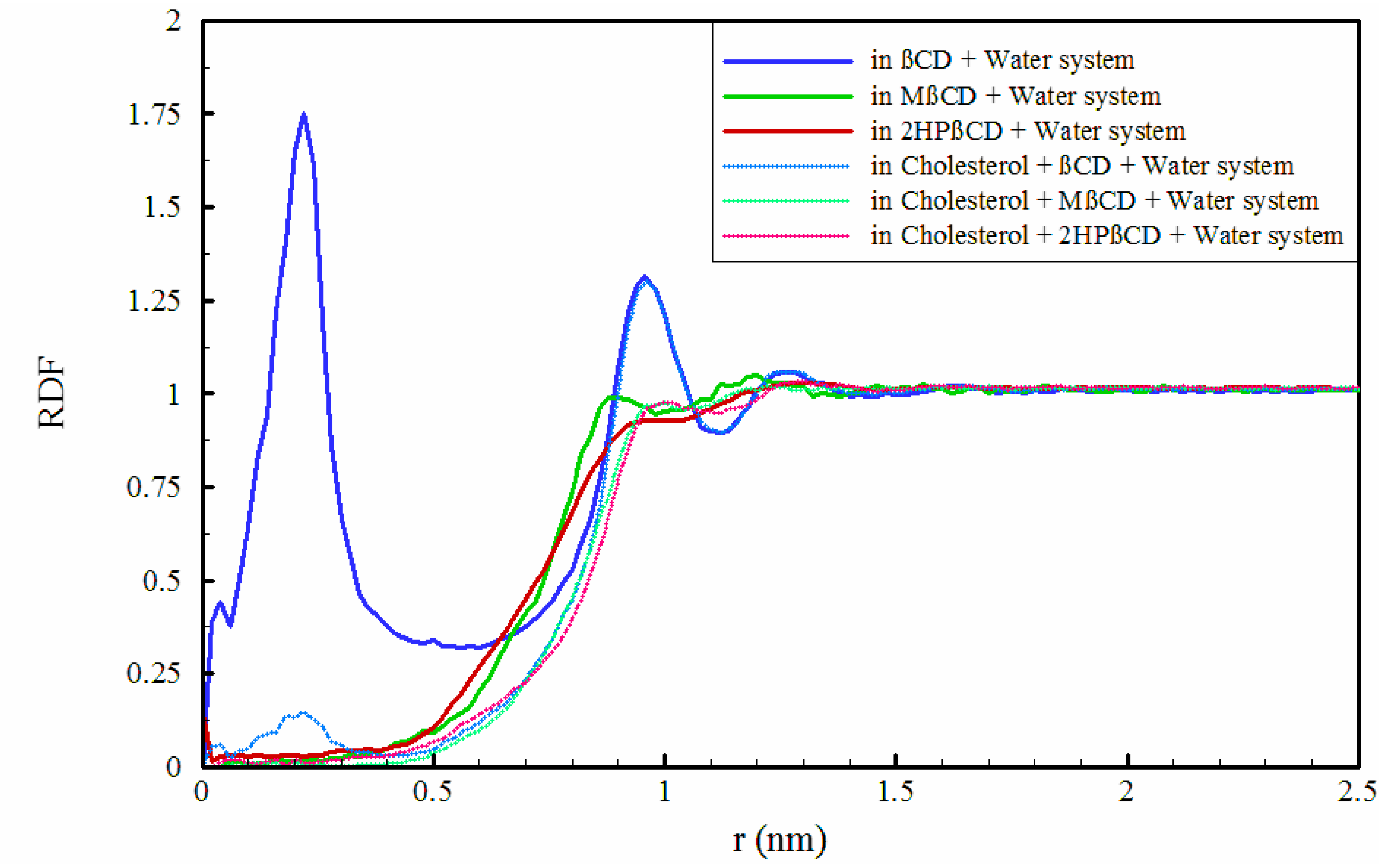

Figure S7: Radial distribution function (RDF) of water around CDs in different simulated systems.

Table S4: Conformational Parameters Describing Molecular Arrangement of βCD in different simulated systems[a].

Structural Properties / Systems	A_{PHR} (nm^2)	A_{MID} (nm^2)	A_{SHR} (nm^2)	Ω_{O1}	Ω_{O2}	Ω_{O6}	h_{12} (nm)	h_{16} (nm)	V_C (nm^3)
**Replica-1**	**1.12**	**0.84**	**1.31**	**0.98**	**0.99**	**0.89**	**0.23**	**0.33**	**0.57**
**Replica-2**	**1.18**	**0.84**	**1.29**	**0.99**	**0.99**	**0.89**	**0.23**	**0.33**	**0.57**
**Replica-3**	**1.18**	**0.84**	**1.28**	**0.99**	**0.99**	**0.88**	**0.23**	**0.33**	**0.57**
**Replica-4**	**1.13**	**0.83**	**1.31**	**0.99**	**0.99**	**0.87**	**0.23**	**0.33**	**0.57**

[a]All results were obtained from the last 10 % of the simulation time.

Table S5: Conformational Parameters Describing Molecular Arrangement of MβCD in different simulated systems[a].

Structural Properties / Systems	A_{PHR} (nm^2)	A_{MID} (nm^2)	A_{SHR} (nm^2)	Ω_{O1}	Ω_{O2}	Ω_{O6}	h_{12} (nm)	h_{16} (nm)	V_C (nm^3)
**Replica-1**	**1.33**	**0.84**	**1.27**	**0.97**	**0.98**	**0.87**	**0.22**	**0.34**	**0.60**
**Replica-2**	**1.32**	**0.84**	**1.27**	**0.96**	**0.98**	**0.84**	**0.22**	**0.34**	**0.59**
**Replica-3**	**1.26**	**0.84**	**1.30**	**0.96**	**0.98**	**0.86**	**0.22**	**0.34**	**0.59**
**Replica-4**	**1.33**	**0.84**	**1.27**	**0.96**	**0.98**	**0.86**	**0.22**	**0.34**	**0.60**

[a]All results were obtained from the last 10 % of the simulation time.

Table S6: Conformational Parameters Describing Molecular Arrangement of 2HPβCD in different simulated systems[a].

Structural Properties / Systems	A_{PHR} (nm^2)	A_{MID} (nm^2)	A_{SHR} (nm^2)	Ω_{O1}	Ω_{O2}	Ω_{O6}	h_{12} (nm)	h_{16} (nm)	V_C (nm^3)
**Replica-1**	**1.35**	**0.85**	**1.21**	**0.97**	**0.97**	**0.78**	**0.23**	**0.32**	**0.58**
**Replica-2**	**1.35**	**0.85**	**1.21**	**0.97**	**0.97**	**0.81**	**0.23**	**0.32**	**0.58**
**Replica-3**	**1.34**	**0.85**	**1.21**	**0.97**	**0.97**	**0.78**	**0.23**	**0.32**	**0.58**
**Replica-4**	**1.34**	**0.85**	**1.21**	**0.98**	**0.98**	**0.84**	**0.23**	**0.32**	**0.58**

[a]All results were obtained from the last 10 % of the simulation time.

Calculation methods for area and volume of cavity

The area of CDs cavity was calculated by the following equation:

$$A = \frac{\pi}{7}\sum_{i=1}^{7} r_i^2$$

Where r_i is the distance between each hydroxyl group and the center of O1 atoms, and the hydroxyl groups at 6- and 3-positions are used for representing the cavity area of primary and secondary hydroxyl rims, respectively.

The CD cavity has a shape that resembles a conical hourglass. As a result, we can approximate its volume by combining the volumes of the truncated cones located at the top and bottom of the cavity, as shown below.

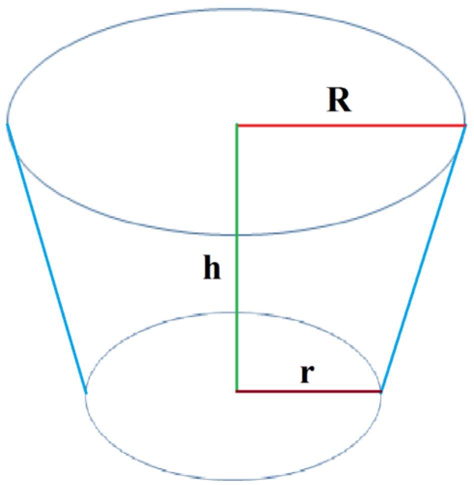

One can determine the volume of a truncated cone with a small radius "r", a large radius "R", and a height "h" by considering its geometry as follows:

$$Vc = \frac{1}{3}\pi h(r^2 + r \times R + R^2)$$

The radius of the O1 rim (Figure 1) was used as the small radius of the cones, while the radius of O2 rim and O6 rim was used as the large radius of the top and the bottom cone, respectively. The height, h, of the cones is sum of h12 and h16 listed in Table 1.

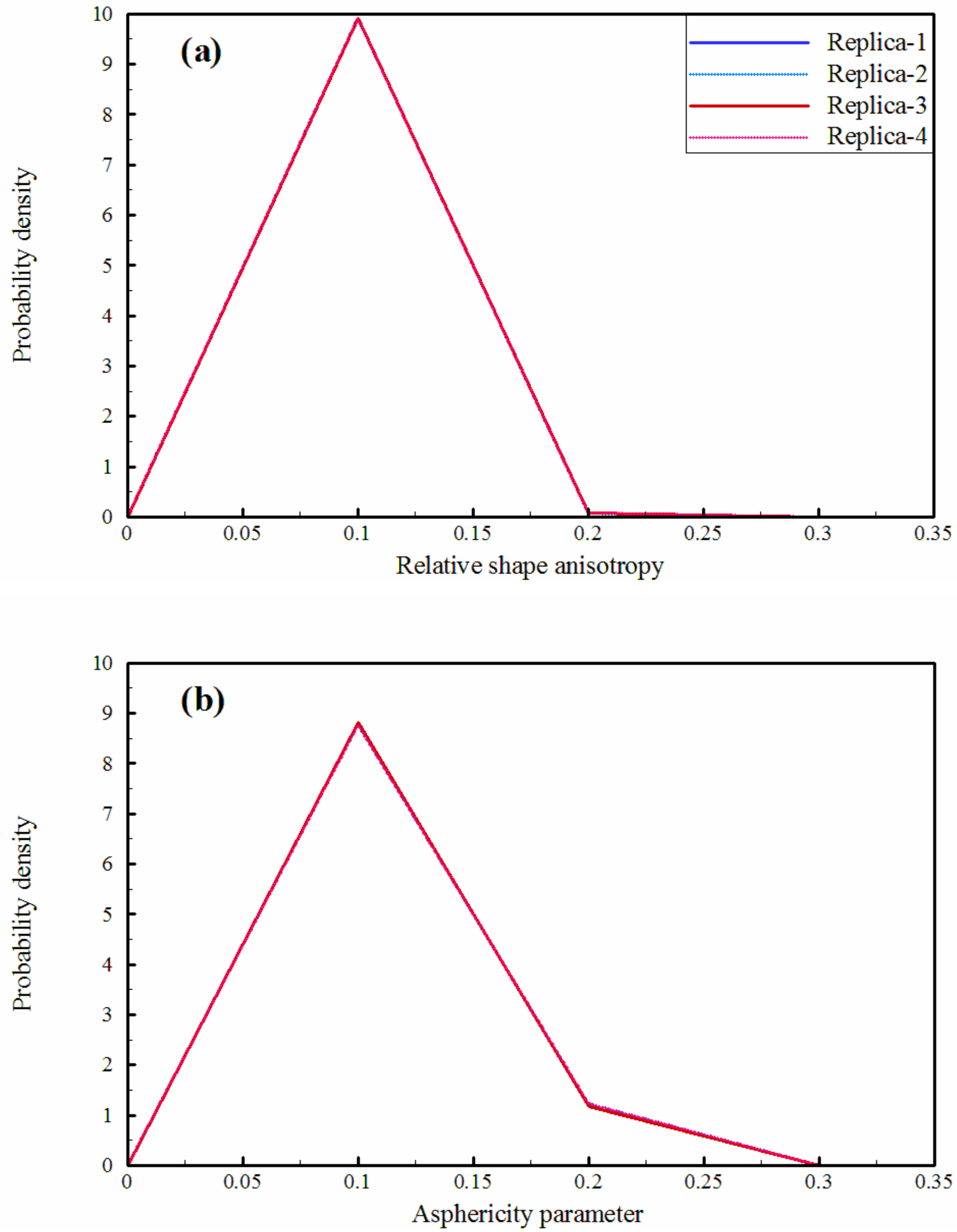

Figure S8: The relative shape anisotropy parameter (a), and the Asphericity parameter (b) of βCD in different simulated systems.

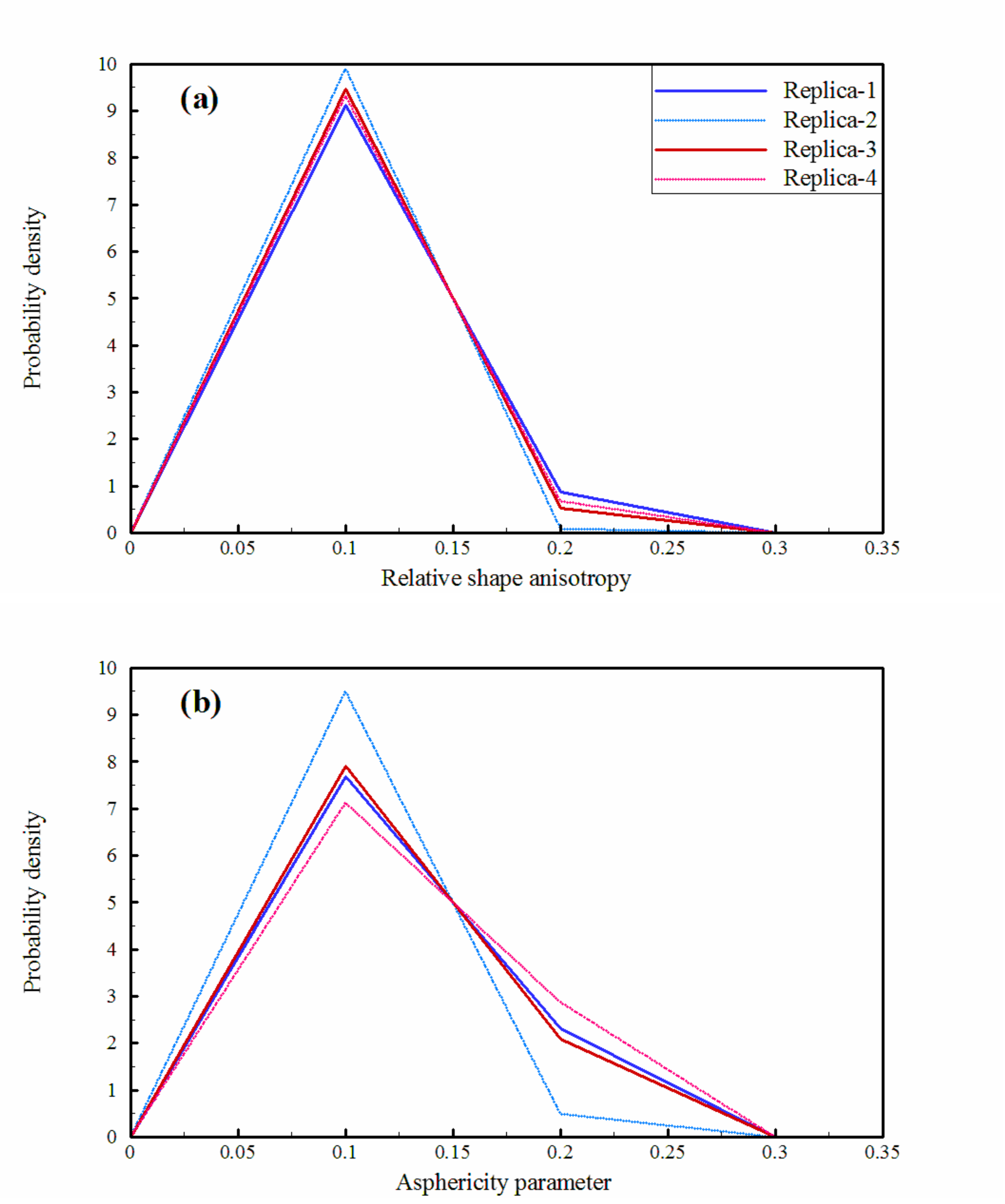

Figure S9: The relative shape anisotropy parameter (a), and the Asphericity parameter (b) of MβCD in different simulated systems.

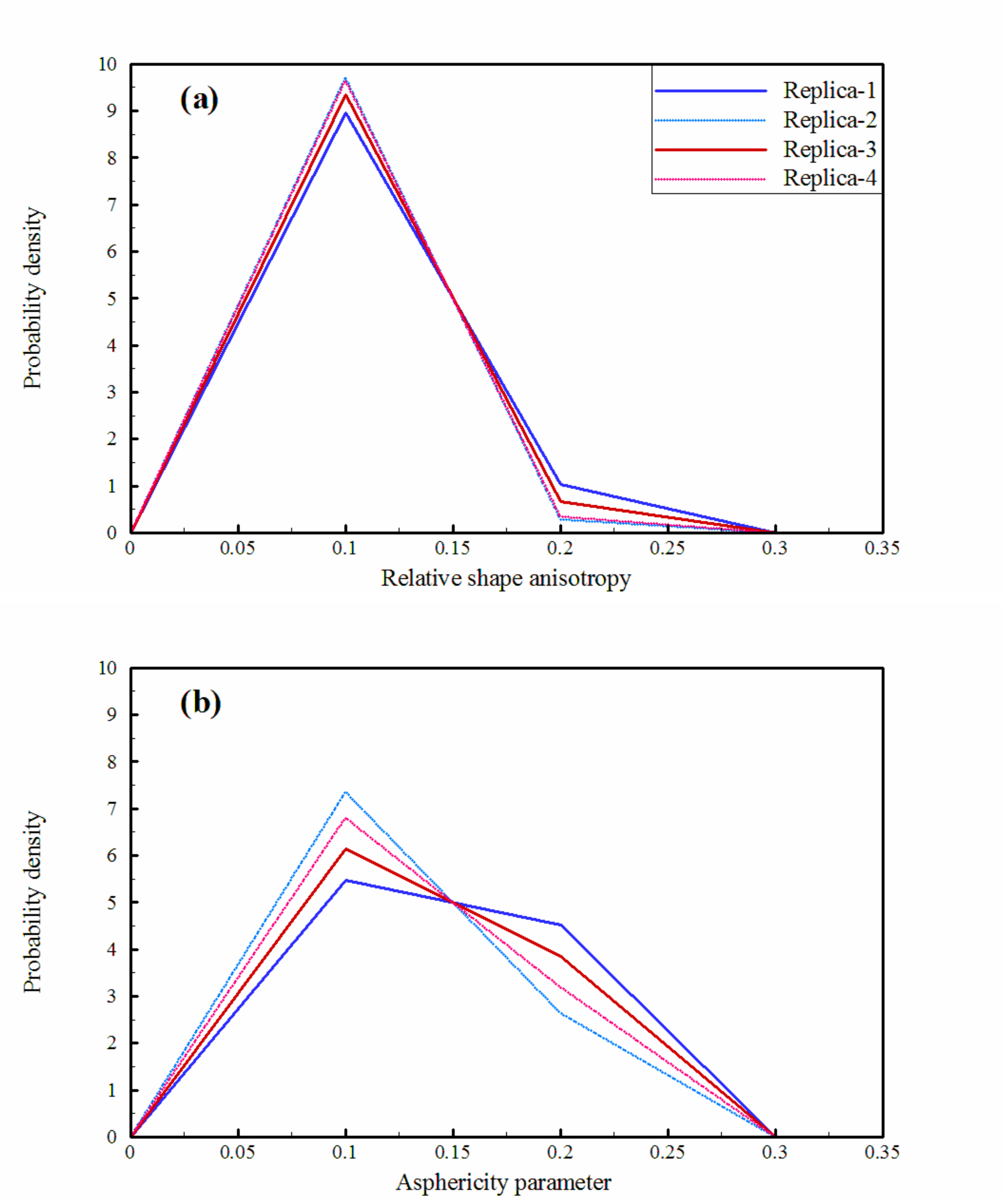

Figure S10: The relative shape anisotropy parameter (a), and the Asphericity parameter (b) of 2HPβCD in different simulated systems.

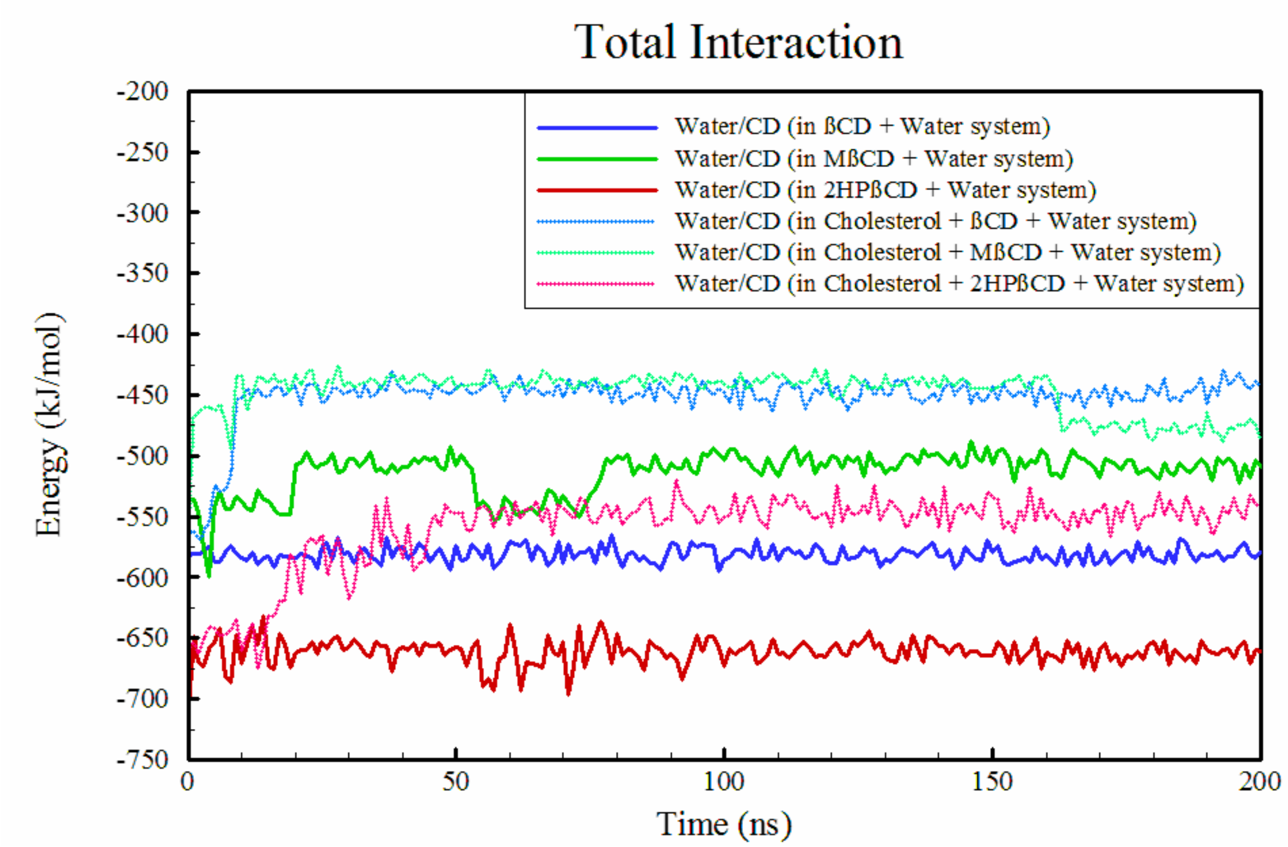

Figure S11: The total interaction energy between water/CDs in different simulated systems.

Table S7: Analyzing of energies in different simulated systems that containing βCD[a].

Energies (kJ/mol) / Simulated systems	vdW between βCD and Cholesterol	Coulombic between βCD and Cholesterol	vdW between βCD and Water	Coulombic between βCD and Water	vdW between Water and Cholesterol	Coulombic between Water and Cholesterol
Water + βCD + Cholesterol	-131.39 (± 0.51)	-0.53 (± 0.04)	-213.78 (± 0.46)	-234.33 (± 1.10)	-79.94 (± 0.56)	-11.63 (± 0.28)
Replica-1	-128.12 (± 0.35)	-0.07 (± 0.03)	-212.31 (± 0.16)	-234.07 (± 1.10)	-80.91 (± 0.14)	-12.23 (± 0.28)
Replica-2	-133.58 (± 0.74)	-0.59 (± 0.04)	-212.98 (± 0.27)	-233.00 (± 1.30)	-79.05 (± 0.42)	-11.30 (± 0.25)
Replica-3	-132.95 (± 0.69)	-0.54 (± 0.02)	-213.69 (± 0.75)	-232.33 (± 2.10)	-79.33 (± 0.33)	-11.64 (± 0.19)
Replica-4	-128.71 (± 0.12)	-0.07 (± 0.01)	-212.69 (± 0.39)	-233.22 (± 1.40)	-81.16 (± 0.34)	-11.95 (± 0.24)

[a]All results were obtained from the last 10 % of the simulation time.

Table S8: Analyzing of energies in different simulated systems that containing MβCD[a].

Energies (kJ/mol) / Simulated systems	vdW between MβCD and Cholesterol	Coulombic between MβCD and Cholesterol	vdW between MβCD and Water	Coulombic between MβCD and Water	vdW between Water and Cholesterol	Coulombic between Water and Cholesterol
Water + MβCD + Cholesterol	-157.72 (± 0.71)	0.08 (± 0.03)	-285.97 (± 0.54)	-191.67 (± 1.50)	-66.83 (± 0.39)	-12.84 (± 0.28)
Replica-1	-160.40 (± 0.95)	-0.04 (± 0.03)	-286.64 (± 0.40)	-189.86 (± 0.92)	-67.58 (± 0.38)	-11.97 (± 0.31)
Replica-2	-161.14 (± 1.00)	0.06 (± 0.05)	-285.52 (± 0.53)	-191.67 (± 1.20)	-66.57 (± 0.23)	-12.10 (± 0.22)
Replica-3	-153.33 (± 0.65)	-0.35 (± 0.06)	-283.33 (± 0.81)	-189.10 (± 1.30)	-69.75 (± 0.18)	-12.40 (± 0.25)
Replica-4	-161.36 (± 1.30)	0.07 (± 0.07)	-285.92 (± 0.39)	-189.62 (± 1.20)	-66.55 (± 0.42)	-12.34 (± 0.18)

[a]All results were obtained from the last 10 % of the simulation time.

Table S9: Analyzing of energies in different simulated systems that containing 2HPβCD[a].

Energies (kJ/mol) / Simulated systems	vdW between 2HPβCD and Cholesterol	Coulombic between 2HPβCD and Cholesterol	vdW between 2HPβCD and Water	Coulombic between 2HPβCD and Water	vdW between Water and Cholesterol	Coulombic between Water and Cholesterol
Water + 2HPβCD + Cholesterol	-159.24 (± 0.81)	-0.59 (± 0.04)	-304.48 (± 0.96)	-241.44 (± 1.00)	-62.42 (± 0.22)	-11.20 (± 0.28)
Replica-1	-159.67 (± 0.40)	-0.63 (± 0.08)	-304.84 (± 1.10)	-241.51 (± 0.69)	-62.61 (± 0.28)	-11.43 (± 0.11)
Replica-2	-158.35 (± 0.56)	-0.55 (± 0.01)	-304.56 (± 1.60)	-239.93 (± 2.10)	-62.89 (± 0.58)	-11.36 (± 0.21)
Replica-3	-159.06 (± 0.27)	-0.59 (± 0.03)	-306.16 (± 0.61)	-240.18 (± 0.90)	-62.48 (± 0.23)	-11.17 (± 0.24)
Replica-4	-158.43 (± 1.10)	-0.81 (± 0.12)	-306.33 (± 1.40)	-241.43 (± 2.30)	-63.48 (± 0.85)	-11.18 (± 0.29)

[a]All results were obtained from the last 10 % of the simulation time.

Table S10: Average number of different hydrogen bonds in the βCD simulated systems[a].

System \ H-bond	Between water and Cholesterol (lifetime)	Between water and βCD (lifetime)	Between Cholesterol and βCD (lifetime)	Between βCD and βCD (lifetime)
**Replica-1**	**1.03 (±10.79)**	**15.13 (±10.76)**	**0.00 (±0.00)**	**7.36 (±35.22)**
**Replica-2**	**0.98 (±10.94)**	**15.08 (±10.67)**	**0.00 (±0.00)**	**7.58 (±36.52)**
**Replica-3**	**0.97 (±10.54)**	**15.02 (±10.73)**	**0.00 (±0.00)**	**7.57 (±37.07)**
**Replica-4**	**0.98 (±10.71)**	**15.18 (±10.80)**	**0.00 (±0.00)**	**7.40 (±35.85)**
**Water + Cholesterol**	**1.02 (±10.58)**	-----	-----	-----

[a]All results were obtained from the last 10 % of the simulation time.

Table S11: Average number of different hydrogen bonds in the MβCD simulated systems[a].

System \ H-bond	Between water and Cholesterol (lifetime)	Between water and MβCD (lifetime)	Between Cholesterol and MβCD (lifetime)	Between MβCD and MβCD (lifetime)
**Replica-1**	**1.03 (±10.69)**	**10.31 (±11.60)**	**0.00 (±0.00)**	**4.30 (±46.66)**
**Replica-2**	**1.01 (±10.83)**	**10.30 (±11.67)**	**0.00 (±0.00)**	**4.41 (±47.59)**
**Replica-3**	**1.07 (±10.58)**	**10.37 (±11.45)**	**0.00 (±0.00)**	**4.24 (±43.87)**
**Replica-4**	**1.03 (±10.65)**	**10.31 (±11.61)**	**0.00 (±0.00)**	**4.47 (±48.97)**
**Water + Cholesterol**	**1.02 (±10.58)**	-----	-----	-----

[a]All results were obtained from the last 10 % of the simulation time.

Table 12: Average number of different hydrogen bonds in the 2HPβCD simulated systems[a].

System \ H-bond	Between water and Cholesterol (lifetime)	Between water and 2HPβCD (lifetime)	Between Cholesterol and 2HPβCD (lifetime)	Between 2HPβCD and 2HPβCD (lifetime)
**Replica-1**	**0.98 (±10.82)**	**15.66 (±10.82)**	**0.010 (±12.50)**	**8.10 (±33.59)**
**Replica-2**	**0.99 (±10.65)**	**15.70 (±10.96)**	**0.010 (±15.71)**	**8.33 (±35.19)**
**Replica-3**	**0.98 (±10.27)**	**15.88 (±10.94)**	**0.002 (±10.00)**	**8.41 (±35.55)**
**Replica-4**	**0.96 (±10.60)**	**15.95 (±10.90)**	**0.05 (±17.27)**	**8.14 (±33.82)**
**Water + Cholesterol**	**1.02 (±10.58)**	-----	-----	-----

[a]All results were obtained from the last 10 % of the simulation time.

Table S13: The number of acceptors and donors involved in hydrogen bonding in this study.

CDs	***Number of acceptors***	***Number of donors***
βCD	35	21
MβCD	35	7
2HPβCD	41	21
